# Mistranslation Reduces Mutation Load in Evolving Proteins through Negative Epistasis with DNA Mutations

**DOI:** 10.1093/molbev/msab206

**Published:** 2021-07-13

**Authors:** Jia Zheng, Ning Guo, Andreas Wagner

**Affiliations:** 1 Department of Evolutionary Biology and Environmental Studies, University of Zurich, Zurich, Switzerland; 2 Swiss Institute of Bioinformatics, Quartier Sorge-Batiment Genopode, Lausanne, Switzerland; 3 Wallisellen, Zurich, Switzerland; 4 The Santa Fe Institute, Santa Fe, NM, USA

**Keywords:** molecular evolution, phenotypic mutations, mistranslation, negative epistasis, mutation load

## Abstract

Translational errors during protein synthesis cause phenotypic mutations that are several orders of magnitude more frequent than DNA mutations. Such phenotypic mutations may affect adaptive evolution through their interactions with DNA mutations. To study how mistranslation may affect the adaptive evolution of evolving proteins, we evolved populations of green fluorescent protein (GFP) in either high-mistranslation or low-mistranslation *Escherichia coli* hosts. In both hosts, we first evolved GFP under purifying selection for the ancestral phenotype green fluorescence, and then under directional selection toward the new phenotype yellow fluorescence. High-mistranslation populations evolved modestly higher yellow fluorescence during each generation of evolution than low-mistranslation populations. We demonstrate by high-throughput sequencing that elevated mistranslation reduced the accumulation of deleterious DNA mutations under both purifying and directional selection. It did so by amplifying the fitness effects of deleterious DNA mutations through negative epistasis with phenotypic mutations. In contrast, mistranslation did not affect the incidence of beneficial mutations. Our findings show that phenotypic mutations interact epistatically with DNA mutations. By reducing a population’s mutation load, mistranslation can affect an important determinant of evolvability.

## Introduction

DNA mutations are the raw material of adaptive evolution (Halligan and Keightley 2009; Olson-Manning et al. 2012). The fitness effect of any one such mutation can depend on other mutations that it occurs with. Such nonadditive or epistatic interactions between mutations are pervasive in proteins (Bershtein et al. 2006; Sarkisyan et al. 2016), and they can profoundly affect adaptive evolution (Salverda et al. 2011; Bank et al. 2016). For example, consider two beneficial mutations whose interaction shows positive epistasis, that is, the two mutations together lead to a fitter phenotype than expected from adding their individual fitness effects. Such epistasis has been observed when a mutation that brings forth a new protein function but destabilizes the protein co-occurs with a mutation that stabilizes the protein (Bloom et al. 2006; Salverda et al. 2017). Positive epistasis can speed the spreading of beneficial mutations and thus promote adaptive evolution (Weinreich et al. 2006; Salverda et al. 2011; Zheng et al. 2019).

Not all mutations that display positive epistasis with beneficial DNA mutations themselves need to be DNA mutations. They can also be phenotypic mutations that occur during protein synthesis (Goldsmith and Tawfik 2009). Mistranslation, the erroneous incorporation of amino acids into proteins by a ribosome, is an abundant source of such phenotypic mutations, because translational errors are several orders of magnitude more frequent than DNA mutations (Kramer and Farabaugh 2007; Drummond and Wilke 2008). Consider a translated protein molecule that carries a beneficial DNA mutation. If a second mutation caused by mistranslation has a positive epistatic interaction with the genetic mutation, the result will be a greater fitness increase than expected when each mutation acts on its own. As a result, such a phenotypic–DNA mutant combination may spread in a population and serve as a “stepping stone” to further adaptive DNA mutations. In an extreme case, if both the DNA and phenotypic mutations are neutral or deleterious on their own but improve fitness when they occur together, a theoretically predicted phenomenon called the “look-ahead” effect may occur (Whitehead et al. 2008). This phenomenon could promote adaptive evolution by augmenting the beneficial effects of DNA mutations.

Although beneficial mutations are most important for adaptive evolution, they are much less abundant than deleterious mutations. The reason is that most DNA mutations destabilize proteins and thus damage protein function (Bershtein et al. 2006; Eyre-Walker and Keightley 2007; Peris et al. 2010; Rockah-Shmuel et al. 2015; Sarkisyan et al. 2016; Wrenbeck et al. 2017). In addition, deleterious mutations that accumulate during protein evolution can gradually decrease protein stability and foldability (Bershtein et al. 2006; Bershtein and Tawfik 2008; Zheng et al. 2020), render proteins more sensitive to mutations, and thus further increase the incidence of deleterious mutations. When two or more deleterious mutations occur together, they usually reduce fitness to a greater extent than expected from their additive effects on fitness, a phenomenon also known as negative epistasis (Bershtein et al. 2006; Melamed et al. 2013; Olson et al. 2014; Bank et al. 2015; Sarkisyan et al. 2016; Gonzalez and Ostermeier 2019). In consequence, the accumulation of deleterious DNA mutations can hinder adaptive evolution because it promotes the deterioration of folding stability and thus reduces the penetrance of neofunctionalizing mutations, that is, mutations that endow a protein with a new function (Masel 2006; Bershtein and Tawfik 2008; Zheng et al. 2020).

Any mechanism that can help eliminate deleterious DNA mutations may facilitate adaptive evolution. For example, DNA recombination associated with sexual reproduction can promote adaptation by separating beneficial mutations from deleterious mutations through recombination (Mcdonald et al. 2016). Strong selection can promote adaptive evolution by purging deleterious mutations (Masel 2006; Bershtein and Tawfik 2008; Zheng et al. 2020). Under purifying selection, negative epistasis can help eliminate deleterious mutations by amplifying the deleterious effects of individual mutations. Such amplification might also be achieved via phenotypic mutations caused by mistranslation. In other words, phenotypic mutations might display negative interactions with DNA mutations and thus amplify their deleterious effects. In this way, mistranslation might promote the elimination of deleterious DNA mutations and help natural selection purge them from a population through purifying selection. By enhancing the purging of harmful mutations, it might also enhance a population’s evolvability—the ability to evolve new and adaptive phenotypes—under directional selection for a new phenotype.

Prior theoretical research proposed that phenotypic mutations can speed adaptive evolution by forming epistatically interacting phenotypic–genotypic mutation pairs (Whitehead et al. 2008), but we lack pertinent empirical evidence for such epistasis. Although previous studies have shown that phenotypic mutations caused by mistranslation can affect adaptive evolution (Giacomelli et al. 2007; Javid et al. 2014; Bratulic et al. 2015; Fan et al. 2015; Yanagida et al. 2015; Samhita et al. 2020), whether they did so through their positive or negative epistatic interactions with DNA mutations is unknown. For example, prior work showed that mistranslation can help purge deleterious mutations on the evolution of antibiotic-resistance enzyme β-lactamase (Bratulic et al. 2017). However, antibiotic resistance phenotypes are not ideally suited to detect epistasis, because it is hard to quantify such phenotypes with high precision and for individual cells. Instead, most pertinent work uses semiquantitative measures of antibiotic resistance that are determined as population averages (Bratulic et al. 2017; Salverda et al. 2017). In consequence, previous work was unable to ascertain whether epistasis is the genetic mechanism by which elevated mistranslation helps purge deleterious mutations. In addition, mistranslation can affect the entire proteome, and can thus pose a challenge to disentangle its proteome-wide effects from effects on individual proteins.

To overcome these limitations, we here use a green fluorescent protein (GFP) study system, which enabled us to characterize phenotypes of evolving populations at single-cell resolution through fluorescence-activated cell sorting (FACS). This study system enabled us to detect whether phenotypic mutations epistatically interact with DNA mutations. In addition, GFP is not native to *Escherichia coli*, which minimizes its interference with the native *E. coli* proteome (Bratulic et al. 2015, 2017). More specifically, we subjected populations of GFP to multiple rounds of directed evolution (Materials and Methods) in either high-mistranslation or low-mistranslation *E. coli* hosts. Our evolution experiment comprised two phases. In phase I, we subjected GFP to purifying selection aimed at maintaining its ancestral green fluorescence phenotype. Our main goal was to study how mistranslation may affect a population’s load of DNA mutations. In phase II, we subjected GFP populations from the end of phase I to directional selection for the derived (“new”) phenotype of yellow fluorescence, to study how mistranslation may affect protein evolvability. We then analyzed the genetic changes that occurred in both populations in molecular detail by high-throughput sequencing.

## Results

### Elevated Mistranslation Reduces Mutation Load through Negative Epistasis between Phenotypic and DNA Mutations

**Fig. 1.**
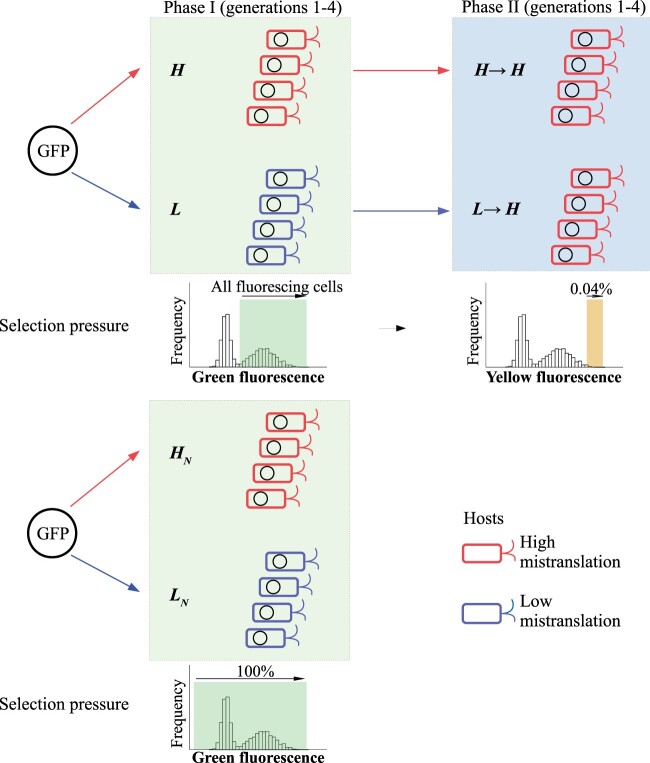
Experimental evolution of GFP. In phase I, we subjected four replicate populations of GFP expressed in the high-mistranslation host (red; populations *H*) or the low-mistranslation host (blue; populations *L*) to four generations of directed evolution under purifying selection for the native green fluorescence (upper left panel), allowing cells whose fluorescence intensities are higher than 99.9% of cells that do not express GFP to survive (green shading, see Materials and Methods; λ_ex_ = 405 nm and λ_em_ = 525 ± 25 nm). We also evolved four replicate GFP populations in the high-mistranslation and low-mistranslation hosts under no selection, by allowing all cells regardless of their fluorescence to survive (populations *H*_N_ and *L*_N_, lower left panel). In phase II, we subjected the *L* and *H* populations from the end of phase I to four further generations of strong directional selection for yellow fluorescence under high-mistranslation (designated as populations *H*→*H* and *L*→*H*, upper right), allowing only the top 0.04% of cells to survive (λ_ex_ = 488 nm and λ_em_ = 530 ± 15 nm, see Materials and Methods). After each generation, we isolated plasmids from the selected cells, and used these plasmids as templates for the next mutation-selection cycle.

We performed directed GFP evolution in an *E. coli* MG1655 strain with a high mistranslation rate that is caused by a mutated ribosomal protein *S4* gene. The mutation causes a substantial increase of missense, read-through, and frameshift errors during protein synthesis (Kramer and Farabaugh 2007; Fan et al. 2015). As a control, we used an *E. coli* MG1655 strain that was isogenic except for a wild-type ribosomal protein *S4* gene, and thus had a low mistranslation rate (see Materials and Methods). For directed evolution, we used a plasmid vector that enables low GFP expression (see Materials and Methods), which minimizes the growth burden of GFP expression in both hosts and causes their GFP expression levels to be similar (supplementary fig. S1, Supplementary Material online). To study whether and how mistranslation reduces mutation load, we subjected four replicate GFP populations to four rounds (“generations”) of directed evolution under purifying selection for the original green fluorescence, by selecting cells fluorescing above background, and did so in both high- and low-mistranslation hosts (populations *H* and *L* in fig. 1; see Materials and Methods). As additional controls, we evolved another four replicate GFP populations in the same way in both high- and low-mistranslation hosts, but under no selection (populations *H*_N_ and *L*_N_ for “Neutral” evolution in fig. 1). We refer to this part of our experiment as phase I. In each generation and in each replicate population of phase I evolution, we used polymerase chain reaction (PCR) mutagenesis to introduce ∼1.6 amino acid changing mutations per green fluorescent protein molecule per generation (supplementary tables S1–S3, Supplementary Material online) and evolved a population of ∼10^6^ GFP variants.

**Fig. 2.**
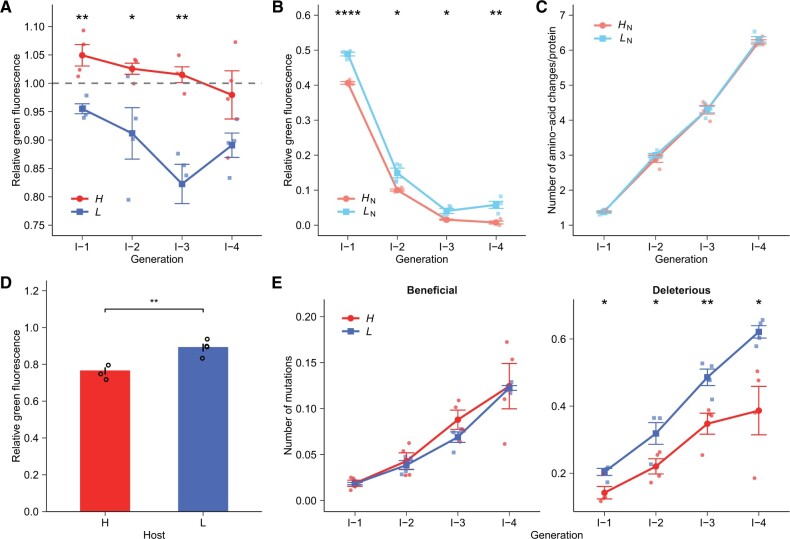
High mistranslation reduces mutation load by helping purge deleterious mutations. (*A*) High mistranslation reduces the mutation load, that is, a decrease in fitness caused by deleterious mutations, under purifying selection. (*B*) High mistranslation leads to a greater fitness decrease under neutral evolution. In panels *A* and *B*, the vertical axes indicate the fluorescence intensity of evolving populations after each generation (horizontal axes) of phase I evolution relative to ancestral GFP populations. (*C*) Number of amino acid changes per protein sequence in populations *H*_N_ or *L*_N_ during phase I evolution. The horizontal axis indicates time (generations of directed evolution), and the vertical axis indicates the number of amino acid changing mutations per protein sequence. (*D*) Deleterious mutations lead to a greater fitness decrease in the high-mistranslation host than in the low-mistranslation host. We retransformed the isolated plasmids of populations *L* at the end of phase I into low-mistranslation (*L*) and high-mistranslation (*H*) hosts, and then determined their residual fluorescence relative to ancestral GFP in the corresponding low-mistranslation and high-mistranslation hosts, respectively (vertical axis). (*E*) Mistranslation reduces the accumulation of deleterious mutations. Each panel shows the evolutionary dynamics of beneficial (left) and deleterious (right) mutations in evolving populations *H* (red) and *L* (blue) during phase I evolution. Error bars represent 1 SD, based on four replicate populations (shown as small symbols). We performed one-sided *t*-tests to determine whether the relative green fluorescence (panels *A*–*D*) or the number of beneficial or deleterious mutations per protein molecule (panel *E*) was significantly different between populations *H* and *L* or between populations *H*_N_ and *L*_N_. *P <* *0.05, **P <* *0.01, ****P <* *0.0001.

Under weak purifying selection, which removes nonfluorescing variants, populations *H* retained higher relative green fluorescence than populations *L* during each generation of phase I evolution (fig. 2*A*). This observation supports our hypothesis that high mistranslation reduces mutation load. In contrast, populations *H*_N_ retained significantly lower relative green fluorescence than populations *L*_N_ during each generation of phase I evolution (*P *<* *0.05, one-sided *t*-tests; fig. 2*B*). Specifically, the relative green fluorescence of populations *H*_N_ was more than 1.2-fold lower than that of populations *L*_N_ throughout phase I evolution. To understand the genetic basis of this difference, we used single-molecule real-time (SMRT) sequencing to genotype ∼500–2,000 protein variants of each replicate population at the end of evolution (supplementary table S1, Supplementary Material online). We then determined the numbers of amino acid mutations in populations *H*_N_ and *L*_N_ in each generation. We found that populations *H*_N_ accumulated almost the same number of mutations per protein molecule as populations *L*_N_ in each generation of phase I (fig. 2*C*). This indicates that the same number of mutations per protein molecule will lead to a greater fitness decrease in high-mistranslation than in low-mistranslation hosts. (We use fluorescence intensity as a proxy for fitness, because selection acts specifically on fluorescence in our experiments.) To calculate the relative green fluorescence of populations *H*_N_ and *L*_N_ (fig. 2*B*), it is most appropriate to divide their absolute fluorescence intensity by that of ancestral GFP in the corresponding high-mistranslation and low-mistranslation hosts. This normalization accounts for differences in the incidence of phenotypic mutations between the two host strains. In other words, if DNA mutations interacted additively with phenotypic mutations, this normalization would ensure that the fitness of *H*_N_ and *L*_N_ mutations would be equal. In contrast, *H*_N_ populations showed a greater fitness decrease than *L*_N_ populations (fig. 2*B*), which implies negative epistasis between phenotypic mutations and deleterious DNA mutations. To further validate this conclusion, we retransformed the isolated plasmids of populations *L* at the end of phase I into low-mistranslation and high-mistranslation hosts, and then compared their relative fluorescence with their ancestors in the corresponding hosts. They retained significantly lower relative fluorescence in high-mistranslation hosts than in low-mistranslation hosts (*P *<* *0.01, one-sided *t*-test; fig. 2*D*), which underscores that high mistranslation causes deleterious DNA mutations to become more deleterious because of negative epistatic interactions between phenotypic mutations and DNA mutations.

The amplification of such deleterious effects may help eliminate deleterious mutations. To find out whether this is the case, we then determined the fitness effect of each mutation by comparing its frequency between populations *H* and *H*_N_ or between populations *L* and *L*_N_ after the last generation of phase I. We considered a mutation beneficial if its frequency was significantly higher in *H* than in *H*_N_ populations, or significantly higher in *L* than in *L*_N_ populations. Conversely, we considered a mutation deleterious if its frequency was significantly lower in *H* than in *H*_N_ populations, or significantly lower in *L* than in *L*_N_ populations. We next characterized the frequency dynamics of beneficial and deleterious mutations in evolving *H* and *L* populations during phase I evolution. Both populations acquired similar numbers of beneficial mutations per protein molecule during each generation of phase I (fig. 2*E*). By contrast, *H* populations accumulated significantly fewer deleterious mutations per YFP molecule in each generation than *L* populations (*P *<* *0.05, one-sided *t*-tests; fig. 2*E*). At the end of phase I, the number of deleterious mutations per YFP molecule was 1.61-fold smaller in *H* than in *L* populations. This analysis confirms that elevated mistranslation helped purge deleterious mutations. In sum, by amplifying the deleterious effects of harmful mutations, elevated mistranslation helps reduce a population’s mutation load, that is, the fitness decrease caused by deleterious mutations.

### Elevated Mistranslation Leads to Modestly Higher Fluorescence during the Evolution of a New Phenotype

**Fig. 3:**
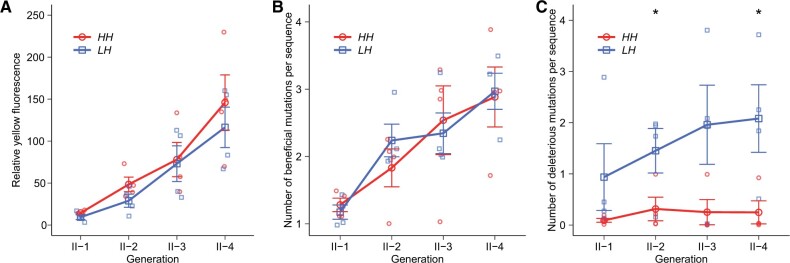
High mistranslation results in modestly higher fluorescence during the evolution of yellow from green fluorescence by improving the efficiency of purging deleterious mutations. (*A*) High-mistranslation populations *H*→*H* evolved slightly higher fluorescence than low-mistranslation populations *L*→*H* in each generation of phase II evolution. The vertical axis indicates yellow fluorescence intensity relative to ancestral GFP for evolving populations in each generation (horizontal axis) of phase II evolution. (*B*) High-mistranslation populations *H*→*H* accumulated beneficial mutations as fast as low-mistranslation populations *L*→*H* during phase II evolution. (*C*) High-mistranslation populations *H*→*H* accumulated fewer deleterious mutations than low-mistranslation populations *L*→*H* during phase II evolution. The horizontal axes show time in generations of directed evolution. The vertical axis indicates the number of beneficial mutations (*B*) or deleterious mutations (*C*) per protein variant. Note that by deleterious mutations we refer to the ten idiosyncratic deleterious mutations (see supplementary fig. S2, Supplementary Material online). We performed one-sided *t*-tests to determine whether the number of beneficial or deleterious mutations per protein variant in each generation was significantly higher in populations *H*→*H* than in populations *L*→*H*. *P <* *0.05. Error bars in panels *A*–*C* represent 1 SD, based on four replicate populations (shown as small symbols).

**Fig. 4.**
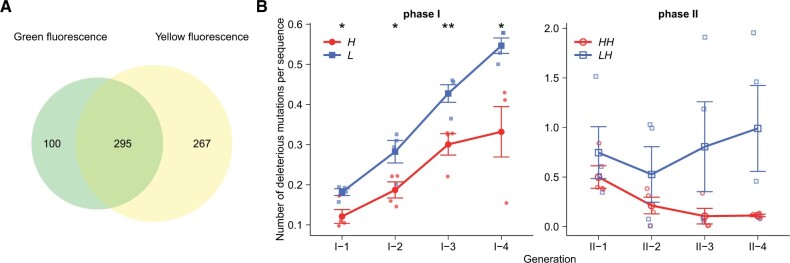
Elevated mistranslation reduces the accumulation of mutations that are deleterious for both ancestral (green) and new (yellow) phenotypes. (*A*) Numbers of mutations with deleterious effects on green fluorescence (green ellipse), yellow fluorescence (yellow ellipse), and both green and yellow fluorescence (overlapping region of green and yellow ellipses). We considered a mutation to be deleterious for green fluorescence if its frequency was significantly lower in *H* than in *H*_N_ populations, or significantly lower in *L* than in *L*_N_ populations after phase I evolution (*P *<* *0.05, one-sided *t*-tests). We considered a mutation to be deleterious for yellow fluorescence if its frequency (*F*_obs_) in populations *H*→*H* or *L*→*H* was significantly lower than the expected frequency (*F*_exp_) after phase II evolution (see details in the main text). (*B*) Numbers of mutations per protein variant that were deleterious for both green and yellow fluorescence during phase I (left) and phase II (right) evolution. The horizontal axes show generations of directed evolution. The vertical axes indicate the numbers of deleterious mutations per protein variant in the corresponding populations. The number of deleterious mutations in populations *L*–*H* did not decrease, possibly because some of these deleterious mutations hitchhiked with beneficial mutations (supplementary fig. S2, Supplementary Material online). We performed one-sided *t*-tests to determine whether the numbers of deleterious mutations per protein variant in each generation were significantly lower in *H* than in *L* populations during phase I evolution and were significantly lower in *H*→*H* than in *L*→*H* during phase II evolution. *P <* *0.05, **P <* *0.01, ****P <* *0.0001.

Because elevated mistranslation reduced mutation load, we suspected that high-mistranslation populations *H* might have an advantage during the evolution of a new phenotype. To test our hypothesis, we conducted a second phase of our experiment (phase II), where we subjected populations *H* and *L* to four additional rounds of directed evolution under selection for the new phenotype yellow fluorescence (fig. 1). We performed phase II evolution in a high-mistranslation host, because this host is more sensitive to deleterious mutations. We reasoned that its sensitivity might make it easier to reveal any differences in evolvability between populations *H* and *L* that are caused by their different mutation loads. In contrast to high-mistranslation hosts, low-mistranslation hosts tolerate deleterious mutations better (fig. 2*D*) and would thus make it more difficult to detect the effects of different mutation loads between populations *H* and *L*. We refer to the resulting populations as *H*→*H* and *L*→*H* populations (fig. 1). We used PCR mutagenesis to introduce 0.59 amino acid changing mutations per protein molecule and per generation into each replicate population (supplementary tables S1–S3, Supplementary Material online), and allowed only the top 0.04% of cells fluorescing in yellow to survive each generation (fig. 1). In each generation of phase II evolution, populations *H*→*H* displayed slightly albeit nonsignificantly higher yellow fluorescence than populations *L*→*H* (*P *>* *0.05, One-sided *t*-tests; fig. 3*A*). Our subsequent analyses would show that this is because high mistranslation helped proteins accumulate significantly fewer deleterious mutations (figs. 2*E*, 3*C*, and 4). In contrast, mistranslation did not strongly affect the number of beneficial mutations per protein molecule (figs. 2*E*, 3*B*, and 4).

### Elevated Mistranslation Helps Purge Deleterious Mutations during Directional Selection for a New Phenotype

To study the genetic changes between *H*→*H* and *L*→*H* populations, we genotyped ∼500–2,000 protein variants per replicate population and per generation (supplementary table S1, Supplementary Material online). We first examined the evolutionary dynamics of amino acid changing mutations and found that different mutations achieved high frequencies in populations *H*→*H* and *L*→*H*. Specifically, we observed 21 mutations that reached a frequency of more than 30% in at least one replicate *H*→*H* or *L*→*H* population at the end of evolution. Among these mutations, only one mutation (C204Y) swept through all replicate *H*→*H* and *L*→*H* populations (supplementary fig. S2, Supplementary Material online). Six further mutations (F65L, K102E, V164A, K167T, I168T, and I168V) achieved high frequencies in at least one replicate *H*→*H* and *L*→*H* population at the evolutionary endpoint. Because these mutations reached high frequencies in both kinds of populations, we call them “general” mutations. The remainder (14 out of 21) mutations achieved high frequencies exclusively in one replicate *H*→*H* or *L*→*H* population. We called these mutations “idiosyncratic.” Three out of 14 idiosyncratic mutations arose in populations *H*→*H*, whereas 11 occurred in populations *L*→*H* (supplementary fig. S2, Supplementary Material online).

Because of the high mutation rates and strong selection we imposed during phase II evolution, we suspected that some of these 21 mutations might be neutral or deleterious and had reached high frequencies by hitchhiking with beneficial mutations (Nash et al. 2005; Chun and Fay 2011; Lang et al. 2013). To find out whether this is the case, we studied the fitness effects of mutations that occurred in populations *H*→*H* and *L*→*H*. To do so, we analyzed the sequence data of evolving populations to identify whether the frequency of each mutant changed beyond what would be expected from mutation pressure alone during phase II evolution.

Specifically, we first determined the frequency *F*_I_ of each mutation in populations *H* and *L* at the end of phase I. We then determined the change *ΔF* in the mutant’s frequency we would expect per generation based on mutation pressure alone. This mutation pressure is caused by the PCR we used for mutagenesis in each generation (see Materials and Methods). To quantify *ΔF*, we analyzed sequence data from libraries of ancestral GFP that we had subjected to our phase II mutagenesis procedure but not to selection (see Materials and Methods). If the mutation was neutral with respect to yellow fluorescence, its expected frequency after four rounds of phase II evolution should be *F*_exp_ = *F*_I_ + 4 × *ΔF*. We then calculated the observed frequency (*F*_obs_) of each mutation from the sequencing data of populations *H*→*H* and *L*→*H* at the end of phase II. We considered a mutation beneficial only if its frequency (*F*_obs_) was significantly higher than the expected frequency (*F*_exp_). Conversely, we considered a mutation deleterious if its frequency (*F*_obs_) was significantly lower than the expected frequency (*F*_exp_).

We first applied this strategy to determine which of the 21 mutations that achieved a frequency exceeding 30% were beneficial. Four out of seven general mutations (F65L, V164A, I168T, and C204Y) met this criterion (*P < 0.05*, One-sided *t*-tests; supplementary fig. S3, Supplementary Material online). In addition, we also included the mutation I168V, which displayed signs of clonal interference with I168T (supplementary figs. S4 and S5, Supplementary Material online) (Lang et al. 2013).

We then compared the number of these five beneficial mutations per GFP molecule between *H*→*H* and *L*→*H* populations during each phase II generation. These numbers were nearly the same between populations *H*→*H* and *L*→*H* (fig. 3*B*). In addition, the same mutations had also accumulated to a similar extent in *H* and *L* populations during phase I evolution (supplementary fig. S6A, Supplementary Material online). This indicates that the mildly higher fluorescence of *H*→*H* populations cannot be attributed to a preferential accumulation of beneficial mutations.

We next hypothesized that the weakly higher fluorescence of populations *H*→*H* might exist, because fewer deleterious mutations accumulated in these populations. Although selection will eliminate strongly harmful mutations, neutral or slightly deleterious mutations may reach a high frequency by hitchhiking with other beneficial mutations. We first focused this analysis on idiosyncratic mutations, which achieved high frequencies in only one replicate population, reasoning that some of them might be slightly deleterious and hitchhike to high frequency with other beneficial mutations. When we analyzed the frequency changes of all 14 idiosyncratic mutations after phase II evolution, we found ten such mutations with lower than expected frequency in either *H*→*H* or *L*→*H* populations (supplementary fig. S3, Supplementary Material online). Because all these differences were statistically significant or marginally significant (supplementary fig. S3, Supplementary Material online), we classified these mutations as deleterious. Importantly, nine of them occurred in *L*→*H* populations, and only one (N106S) occurred in a *H*→*H* population (replicate 1, supplementary fig. S2, Supplementary Material online). We then studied the dynamics of the ten high-frequency deleterious mutations during phase II evolution. Populations *H*→*H* had fewer high-frequency deleterious mutations per protein variant than populations *L*→*H* (fig. 3*C*) in each generation of phase II. Specifically, the numbers of high-frequency deleterious mutations per protein variant in *H*→*H* populations were between 4.61- and 10.24-fold lower than in *L*→*H* populations during phase II evolution (fig. 3*C*). Because *H*→*H* and *L*→*H* populations experienced identical selection strength during phase II evolution, we reasoned that these differences might be a remnant of phase I evolution, where *H* populations might have accumulated fewer such mutations than *L* populations. Indeed, we found that proteins in *H* populations harbored fewer of these mutations during each generation of phase I than in *L* populations (supplementary fig. S6*B*, Supplementary Material online). Specifically, at the end of phase I, the numbers of these mutations per protein molecule were 1.33-fold lower in *H* than in *L* populations. This is consistent with the observation that seven of the ten high-frequency deleterious mutations were also harmful for the original phenotype green fluorescence (supplementary fig. S7, Supplementary Material online). These observations confirm that fewer of these deleterious mutations accumulated in populations *H*→*H* than in populations *L*→*H* during phase II evolution, because high-mistranslation populations *H* had accumulated fewer of them than low-mistranslation populations *L* during phase I evolution.

To further validate our hypothesis that deleterious mutations are central to any advantage mistranslation might have, we used the same approach (fig. 2*E* and supplementary fig. S3, Supplementary Material online) to estimate the fitness effects of *all* mutations, regardless of their final frequency, on both green and yellow fluorescence, and during both phase I and II evolution. This analysis showed that 395 mutations were deleterious for the original phenotype green fluorescence, and 295 of them were also deleterious for yellow fluorescence (fig. 4*A*). This indicates that 74.5% of mutations that were deleterious for the ancestral phenotype were also deleterious for the new phenotype. We called these mutations unconditionally deleterious. They may cause protein truncation or reduce protein foldability and stability, and might thus be deleterious for both phenotypes (Masel 2006; Rockah-Shmuel et al. 2015). We suspected that their elimination during phase I would reduce their accumulation during phase II, which could explain the modestly higher fluorescence of populations *H*→*H* in phase II. To validate this hypothesis, we studied their dynamics during phase I and II evolution. Indeed, throughout phase I evolution high-mistranslation populations *H* harbored significantly fewer unconditionally deleterious mutations per protein molecule than low-mistranslation populations *L* (*P *<* *0.05, one-sided *t*-tests; fig. 4*B*). At the end of phase I, the number of these mutations was 1.65-fold lower in populations *H* than in populations *L*. When subject to directional evolution toward yellow fluorescence in phase II, populations *H*→*H* also harbored fewer such mutations per protein variant in each generation than populations *L*→*H* (fig. 4*B*). These observations further confirmed that the purging of deleterious mutations by mistranslation is important during adaptive evolution.

## Discussion

Most mutations in proteins are deleterious (Eyre-Walker and Keightley 2007; Rockah-Shmuel et al. 2015; Sarkisyan et al. 2016; Wrenbeck et al. 2017). Under purifying selection, such mutations may accumulate in a population, which increases the population’s mutation load, and can even drive the population to extinction (Lynch and Gabriel 1990; Lynch et al. 1993; Agrawal and Whitlock 2012). The reduction of this mutation load is thus important if evolving populations are to survive and thrive. In this study, we observed that phenotypic mutations caused by mistranslation can help reduce mutation load. Specifically, elevated mistranslation can help purge deleterious mutations, and thus slow the fitness decay of populations evolving under purifying selection (fig. 2*A*). We also showed that this purging of deleterious mutations (but not the preferential accumulation of beneficial mutations) can result in a modest advantage when a population evolves a novel fluorescent phenotype. Specifically, high-mistranslation populations harbored significantly fewer deleterious mutations per protein molecule than low-mistranslation populations during phase I and phase II evolution but they harbored almost the same number of beneficial mutations per protein molecule (figs. 2*E*, 3, and 4).

One big challenge in studying mistranslation is to distinguish its effects on the evolution of any one protein from its effects on a whole proteome. The reason is that the DNA mutations that are used to study mistranslation, such as the ribosomal protein *S4* mutation in our high mistranslation host, cause proteomic changes and can thus change a host’s growth rate. In previous studies on the antibiotic-resistance enzyme β-lactamase and its evolution under different mistranslation rates (Bratulic et al. 2015, 2017), such interference may have been substantial, because growth rate can strongly affect antibiotic resistance. In this respect, our GFP study system has major advantages. First, GFPs are not native to *E. coli* and thus interfere little with the native *E. coli* proteome. Second, we performed artificial selection based on fluorescence intensity, which is not coupled to cell growth rates. In addition, we expressed fluorescent proteins at low levels in both high- and low-mistranslation strains, which did not change the growth rate of either strain (supplementary fig. S1, Supplementary Material online).

Previous studies indicated that elevated mistranslation might help purge deleterious genetic mutations, because phenotypic mutations have deleterious effects (Bratulic et al. 2015, 2017). Here, we show that this is caused by negative epistatic interactions between genetic mutations and phenotypic mutations (fig. 2*B*–*D*). Negative epistasis between deleterious mutations causes a greater-than-additive fitness decrease and is pervasive in proteins (Bershtein et al. 2006; Melamed et al. 2013; Bank et al. 2015, 2016; Sarkisyan et al. 2016; Gonzalez and Ostermeier 2019). Such negative epistasis amplifies the deleterious effects of genetic mutations and increases the selection coefficients of deleterious phenotypic–genotypic mutant pairs. Specifically, if a deleterious phenotypic mutation A and a deleterious genetic mutations B individually reduce the fitness of ancestral GFP from 1 to 1 − *s*_A_ and to 1 − *s*_B_, where *s*_A_ > 0 and *s*_B_ > 0 are selection coefficients, the fitness of the phenotypic–genotypic pair in the absence of epistasis would equal (1 − *s*_A_) × (1 − *s*_B_). In other words, the selection coefficient for the genetic mutation B in the phenotypic background A would remain *s*_B_. In this case, the occurrence of phenotypic mutation A does not increase the selection coefficient of genetic mutation B, and mutant A would thus not promote the elimination of mutant B. In contrast, if phenotypic mutation A and genetic mutation B show negative epistasis, the fitness of such phenotypic–genotypic pair will decrease to the lower value of (1 − *s*_A_)×(1 − *s*_B_ − *β*). Here, *β > *0 reflects a fitness decrease that is greater than expected under additivity. In other words, the strength of selection against genetic mutation B increases to *S*_B_ + *β* in the phenotypic background A, which thus promotes the elimination of genetic mutation B. (We multiply rather than add the effects of deleterious mutations here to quantify epistasis, because average fitness decays approximately exponentially with the increase in the number of deleterious mutations [Charlesworth 1990; Wilke and Adami 2001; Bershtein et al. 2006].)

In the absence of epistasis between deleterious phenotypic mutations and deleterious genetic mutations, the relative fitness decrease caused by deleterious genetic mutations would not be affected by the incidence of phenotypic mutations in high- and low-mistranslation hosts. In other words, fluorescent proteins evolving in high-mistranslation populations *H*_N_ and low-mistranslation populations *L*_N_ should retain identical residual fluorescence relative to ancestral YFP in the same high- and low-mistranslation hosts after acquiring comparable numbers of genetic mutations. In contrast, we found that populations *H*_N_ retained significantly lower relative fluorescence than populations *L*_N_ in each generation of phase I despite harboring almost the same number of DNA mutations per protein molecule (*P *<* *0.05, one-sided *t*-tests; fig. 2*B*–*D*). This observation confirms the existence of negative epistasis between genetic and phenotypic mutations. The resulting “amplification effect” of phenotypic mutations can reduce the accumulation of deleterious DNA mutations. This prediction is also borne out by our experimental observation that high-mistranslation populations *H* retained higher residual green fluorescence (fig. 2*A*) but lower frequencies of deleterious mutations under purifying selection than low-mistranslation populations *L* in each generation of phase I evolution (fig. 2*E*).

Elevated mistranslation can in principle increase a population’s evolvability in two different ways. The first is by reducing the population’s mutation load, that is, the number or frequency of mutations deleterious for a new phenotype (fig. 4). Most mutations in proteins reduce foldability or stability and cause aggregation or degradation of misfolded proteins (Bershtein et al. 2006; Tokuriki and Tawfik 2009; Rockah-Shmuel et al. 2015; Sarkisyan et al. 2016). In consequence, such mutations will be harmful in any environment and thus are deleterious not only for an ancestral phenotype but also for new phenotypes (Masel 2006). We found that 74.5% of mutations that are deleterious for the original phenotype green fluorescence are also harmful for the new phenotype yellow fluorescence (fig. 4). The accumulation of these mutations during phase I hindered the evolution of the new phenotype during phase II, albeit to a small extent. Elevated mistranslation more efficiently purged these deleterious mutations in phase I and thus prevented their fixation and accumulation in phase II. We note that this advantage caused by elevated mistranslation was modest during phase II evolution. A possible reason is that the eliminated mutations were only weakly deleterious and thus had modestly deleterious effects on fitness (Bratulic et al. 2017).

A second way in which mistranslation could affect evolvability is through beneficial phenotypic mutations. Mistranslated proteins can help create a reservoir of phenotypic variation that might contain adaptive combinations of phenotypic mutants and DNA mutants (Whitehead et al. 2008; Miranda et al. 2013; Javid et al. 2014; Yanagida et al. 2015). For example, elevated mistranslation of RNA polymerase can reduce this enzyme’s susceptibility to the antibiotic rifampicin and thus increase the resistance of *Mycobacterium tuberculosis* to this drug (Javid et al. 2014). More generally, theory predicts that a phenotypic–DNA mutant combination with a large enough selective advantage may spread in a population and serve as a “stepping stone” to highly adaptive DNA mutants (Whitehead et al. 2008). This advantage could be especially great if two deleterious or neutral mutations show reciprocal sign epistasis, that is, if they become beneficial when they occur together. However, we did not observe this so-called “look-ahead” effect. The reason may be that most mutations are deleterious and that negative epistasis is more pervasive than positive epistasis in proteins (Bershtein et al. 2006; Olson et al. 2014; Bank et al. 2015; Rockah-Shmuel et al. 2015; Sarkisyan et al. 2016; Gonzalez and Ostermeier 2019). The likelihood of observing the look-ahead effect may be low for this reason.

Directed evolution experiments usually use high mutation rates, which is necessary to achieve adaptive evolution on laboratory time scales (Bershtein et al. 2006; Salverda et al. 2017). Our experiment is no exception. Its design allowed us to detect substantial differences in both green fluorescence and the frequencies of deleterious mutations between high- and low-mistranslation populations after merely four rounds of phase I evolution under weak purifying selection (fig. 2). However, we observed only a modestly higher relative yellow fluorescence in high-mistranslation populations (fig. 3*A*) during phase II. One possible reason is that we used strong selection in phase II, which efficiently purged the majority of deleterious mutations in both low- and high-mistranslation populations. Under weaker selection for the new phenotype in phase II, the advantage of high-mistranslation populations might be more pronounced. Dissecting the interaction of selection strength and mistranslation during adaptive evolution remains an important task for future work.

In sum, our observations suggest that mistranslation influences adaptive evolution through its effects on deleterious DNA mutations, which it helps purge through their negative epistatic interactions with phenotypic mutations. Translational errors are much more frequent than DNA mutations and can reach up to 10^−3^ per translation event for some *E. coli* codons (Kramer and Farabaugh 2007; Drummond and Wilke 2008; Garofalo et al. 2019; Mordret et al. 2019). For a gene like that encoding GFP (720 nucleotides that encode 239 amino acids), this incidence of mistranslation would imply that ∼7.4% of protein molecules expressed from the gene would harbor one or more misincorporated amino acids. Given such a high incidence of mistranslation, the influence of mistranslation on protein evolution may be substantial. Is mistranslation itself subject to adaptive evolution, because it can help reduce a population’s mutation load? In other words, did evolution balance the physiological costs of mistranslation with the evolutionary benefits caused by a lowered mutation load? To answer this question remains an exciting challenge for future research.

## Materials and Methods

### Strains and Plasmids

We used a high-mistranslation *E. coli* strain which was engineered in a previous study (Bratulic et al. 2015) by replacing the wild-type ribosomal protein *S4* gene of the strain *E. coli* MG1655 with the ribosomal mutant *rpsD12*, which conveys an elevated mistranslation rate (Ballesteros et al. 2001). A matched low-mistranslation strain was engineered by transferring the wild-type ribosomal protein *S4* gene into the MG1655 genetic background to ensure that the high-mistranslation and low-mistranslation strains have the same genetic background except for the ribosomal protein *S4* gene.

We used *E. coli* strain DH5α for cloning and constructing mutation libraries. We used the plasmid pXHO-*eGFP* for directed evolution of GFP (Zheng et al. 2019). This plasmid contains a kanamycin resistance gene, the *pompA* promoter, and a pUC origin of replication (supplementary fig. S8, Supplementary Material online). We constructed this plasmid by replacing the original pSC101 origin of the plasmid pmss201_ompA (Zaslaver et al. 2006) with the pUC replication origin from pBAD202/D-TOPO (Invitrogen, K420201). In this plasmid, the upstream promoter *pompA* helps express the GFP gene *gfp*. We inserted the gene between the restriction sites *Xho*I and *Hind*III, which resulted in low expression levels of GFP in both high-mistranslation and low-mistranslation strains. The *gfp* gene encodes a GFP variant that we had engineered in a previous study (V2M + G66S + 204C + F72C) (Zheng et al. 2019). We chose this variant because it has high green fluorescence but little yellow fluorescence.

### Preparing Electrocompetent Cells

We used glycerol/mannitol step centrifugation as described in a previous study to make electrocompetent cells (Bratulic et al. 2015). Specifically, we inoculated *E. coli* strains into 5 ml SOB medium and grew them overnight at 37 °C and 220 rpm. We then transferred 3 ml overnight culture into 300 ml SOB medium and continued the incubation at 37 °C and 220 rpm until the OD_600_ value had reached 0.4–0.6 (optical path length: 1 cm; ∼2–4 h). We then placed the culture on ice for at least 15 min, centrifuged it at 1,500 × g/4 °C for 15 min to collect cells, and used 60 ml of ice-cold ddH_2_O to resuspend the cells and distribute them into three 50-ml tubes. Subsequently, we slowly added 10 ml ice-cold glycerol/mannitol solution (20% glycerol [w/v] and 1.5% mannitol [w/v]) to the bottom of each tube by using a 10-ml pipette. We collected cells by centrifugation at 1,500 × g and 4 °C for 15 min with acceleration/deceleration set to zero. We aspirated the supernatant and resuspended the pellets using ∼3 ml ice-cold glycerol/mannitol solution. We distributed the resulting suspensions into 1.5 ml precooled tubes, placed them in a dry ice-ethanol bath for around 1 min, and stored them at −80 °C for electroporation.

### Mutagenic PCR

We introduced random mutations into the coding region of GFP by mutagenic PCR, as reported in previous studies (Zaccolo et al. 1996; Bershtein et al. 2006; Bratulic et al. 2015). We used a higher mutation rate in phase I evolution than in phase II evolution (supplementary tables S2 and S3, Supplementary Material online). Specifically, a 50 µl PCR reaction for phase I evolution consisted of 5 ng template plasmid, 2.5 U *Taq* DNA polymerase (M0267L, NEB), 10 µl 10× ThermoPol buffer (M0267L, NEB), 400 µM dNTPs (R0192, Thermo Scientific), 3 µM 8-oxo-GTP/dPTP (Trilink Biotechnologies), and 400 nM primers (misMutaf1- CGTTAAGCAGGAAGAAGTT and misMutar1- GACTGAGCCTTTCGTTTTAT). We executed 25 cycles of PCR as follows for phase I evolution: 95 °C/2 min, 25 cycles of 95 °C/20 s, 46 °C/30 s and 68 °C/50 s, 68 °C/1 min. In phase II evolution, a 50 µl PCR reaction consisted of 15 ng template plasmid, 2.5 U *Taq* DNA polymerase, 10 µl 10×ThermoPol buffer, 400 µM dNTPs, 1 µM 8-oxo-GTP/dPTP, and 400 nM primers (misMutaf1/misMutar1). We executed 20 cycles of PCR as follows for phase II evolution: 95 °C/2 min, 20 cycles of 95 °C/20 s, 46 °C/30 s and 68 °C/50 s, 68 °C/1 min. We used the QIAquick PCR purification kit (Qiagen, Germany) to purify the PCR products. We then added 10 U *Dpn*I (R0176S, NEB) and 20 U *Xho*I/*Hind*III-HF (R0146L/R3104S, NEB) to the purified PCR products and incubated at 37 °C overnight. Subsequently, we used the QIAquick PCR purification kit to purify the digested products.

We used the primers backboneF (CACTCTCGGCATGGACGAGCTGTACAAGT) and backboneR (CCCCGGTGAACAGCTCCTCGCCCTTG) to amplify the vector backbone from the plasmid pXHO-*eGFP* by PCR.

We used high-fidelity Phusion DNA polymerase to reduce the mutation rate during the PCR amplification. A 50 µl PCR reaction consisted of 1 ng template plasmid, 2.5 U Phusion Hot Start II High-Fidelity DNA Polymerase (F-549L, Thermo Scientific), 400 µM dNTPs, 10 µl 5× Phusion HF Buffer, 1.5 µl 100% DMSO, and 400 nM primers backboneF/backboneR. We performed the PCR reaction with the following thermocycler program: 98 °C/30 s, 26 cycles of 98 °C/10 s and 72 °C/60 s, 72 °C/5 min. We used the QIAquick PCR purification kit to purify the PCR products. Subsequently, we added 5 U *Dpn*I and 20 U *Xho*I/*Hind*III-HF at 37 °C overnight and used the QIAquick PCR purification kit to purify the digested products. We then dephosphorylated these products with 5 U Antarctic Phosphatase (M0289S, NEB) and repurified them with the QIAquick PCR purification kit.

We performed the ligation in a 20 µl ligation reaction by mixing ∼50 ng of digested mutagenic PCR products with ∼100 ng digested and dephosphorylated vector backbone, 10 U T4 DNA ligase, and 2 µl 10× ligation buffer (M0202L, NEB). We incubated the ligation reaction at 20–22 °C overnight. After that, we precipitated the ligation product by adding 80 µl ddH_2_O, 1 µl glycogen (R0551, Thermo Scientific), 50 µl 7.5 M ammonium acetate (A2706-100ML, Sigma), and 375 µl ice-cold absolute ethanol. We placed the above mixture at −80 °C for at least 20 min and centrifuged the mixture at 18,000 × g for 20 min. We washed the pellet twice using 800 µl of cold ethanol (70%). We used a concentrator 5301 (Eppendorf) to dry the pellet and then dissolved it in 10 µl ddH_2_O for electroporation.

### Constructing Mutant Libraries

We first transformed the purified ligation products (mutant libraries) into electrocompetent DH5α cells to methylate the plasmid DNA. Such methylation can increase the transformation efficiency in wild-type and error-prone hosts. Specifically, we mixed 50 μl electrocompetent DH5α cells with 4 μl ligation product and transferred the mixture to a 0.2-cm cuvette (EP202, Cell Projects, United Kingdom). We performed electroporation by using a Micropulser electroporator (Bio-Rad) at setting EC3 (15k V/cm). After electroporation, we immediately added 450 µl prewarmed SOC medium. We transferred the suspension into a 10-ml tube and incubated it at 37 °C for 1.5 h with shaking at 220 rpm in a shaking incubator (INFORS HT, Switzerland). We then added 2.5 ml LB medium with 36 μg/ml kanamycin (K1377, Sigma) to continue the incubation overnight. To estimate the library size, we serially diluted 10 μl cell aliquots (in saline) and plated them on LB agar with 20 μg/ml kanamycin. This transformation protocol led to a library size of ∼10^6^ colony-forming units for each replicate population. Subsequently, we used the QIAprep spin miniprep kit (Qiagen, Germany) to extract the plasmids (methylated mutant libraries) from the overnight culture.

### Evolving GFP under Purifying Selection for Green Fluorescence

To transform the methylated mutant libraries into wild-type and error-prone competent cells, we mixed ∼3 ng methylated plasmid with 15 μl wild-type competent cells, or ∼10 ng methylated plasmid with 45 μl error-prone competent cells. We transferred the mixture into a 0.1-cm cuvette (EP201, Cell Projects, United Kingdom) and performed electroporation by using a Micropulser electroporator (Bio-Rad) at setting EC1 (18k V/cm). After electroporation, we immediately added 450 µl prewarmed SOC medium and incubated the culture at 37 °C for 1.5 h with shaking at 220 rpm in a shaking incubator (INFORS HT, Switzerland). We then collected cells by centrifugation at 9,000 × g for 5 min, resuspended cells in 3 ml LB medium with 30 μg/ml of kanamycin, and incubated the culture at 37 °C and 220 rpm. We also sampled 10 μl of the recovered culture and plated a serially diluted aliquot (in saline) on LB agar with 25 μg/ml of kanamycin to estimate library size. This procedure resulted in a library size estimate of ∼10^6^ cells. After ∼22 h of incubation, we mixed 20 μl of the culture with 1 ml of cold phosphate-buffered saline (PBS) buffer in a 5-ml tube for FACS. We used an Aria III cell sorter (BD Biosciences) to sort cells at 4 °C. We performed sorting by using the AmCyan channel (λ_ex_ = 405 nm and λ_em_ = 525 ± 25 nm) and the sort precision of Single Cell Purity. For populations H and L under purifying selection (fig. 1), we collected 10^5^ cells whose fluorescence intensities were higher than 99.9% of cells of negative control populations that do not express GFP. For populations *H*_N_ and *L*_N_ under no selection, we collected 10^5^ cells without considering their fluorescence intensities. We regrew the sorted cells overnight and isolated plasmids from the overnight culture. To avoid the accumulation of mutations in the *E. coli* host genome, we retransformed the isolated plasmid into wild-type and error-prone competent cells as described above. We repeated the sorting process by following the same procedure. We then used plasmids isolated from sorted cells as templates for the next mutation-selection cycle. We repeated the experiments by starting from retransforming methylated mutant libraries into fresh competent cells if cell sorting failed, or if sorted cells completely lost fluorescence. After each generation of evolution, we subjected the isolated variants to SMRT sequencing.

### Evolving GFP under Directional Selection for Yellow Fluorescence

We followed the same procedure as described in “Evolving GFP under Purifying Selection for Green Fluorescence” section to transform the methylated mutant libraries into error-prone competent cells and prepared these evolving populations for sorting (populations *H*→*H* and *L*→*H*; fig. 1). We used an Aria III cell sorter (BD Biosciences) to sort cells at 4 °C, using the FITC channel (λ_ex_ = 488 nm, λ_em_ = 530 ± 15 nm) and the sort precision of Single Cell Purity. We selected 2 × 10^4^ cells in the top 2% of yellow fluorescence intensity (measured as FITC-H) with a sorting speed of ∼10^4^ events/s. We regrew the sorted cells overnight and isolated plasmids from the overnight culture. We retransformed the isolated plasmid into error-prone competent cells as described above. We repeated the sorting process by selecting 10^4^ cells in the top 2% of yellow fluorescence intensity. We then used plasmids isolated from the sorted cells as templates for the next mutation-selection cycle. After each generation of evolution, we subjected the isolated variants to SMRT sequencing.

### Fluorescence Assay Using Flow Cytometry

We noticed that mutations probably occurred in the genome especially of error-prone *E. coli* strains if we grew these strains for many generations. To avoid this problem, we retransformed plasmids isolated from sorted cells from each generation into fresh wild-type or error-prone competent cells as described in “Evolving GFP under Purifying Selection for Green Fluorescence” section. After ∼22 h of incubation, we mixed a 20 μl sample of the culture with 200 μl of cold PBS buffer. We then transferred 20 μl of the mixture to 180 μl cold PBS buffer for a fluorescence assay using flow cytometry. We mixed the resulting suspension thoroughly to measure green fluorescence intensity at the original wavelength (λ_ex_ = 405 nm and λ_em_ = 525 ± 25 nm) and yellow fluorescence at the new wavelength (λ_ex_ = 488 nm and λ_em_ = 530 ± 15 nm). We used a Fortessa cell analyzer (BD Biosciences) with a flow rate of ∼3,000 events/s to measure fluorescence at room temperature. We conducted each assay by analyzing 10^4^ cells for each replicate population. To prevent cell proliferation or death, we put all samples on ice until we had finished the assay.

### Flow Cytometry Data Analysis

We performed flow cytometry data analysis by using FlowJo V10.4.2 (LLC). Specifically, we first selected a homogenous cell population by using forward scatter height (FSC-H) versus side scatter height (SSC-H) density plots. We then excluded doublets by using side scatter area (SSC-A) versus side scatter height density plots. We calculated the mean fluorescence intensity of each biological replicate by using the resulting filtered data.

### SMRT Sequencing

To perform SMRT sequencing, we used two-step PCRs to barcode GFP variants of each population during each generation of evolution, as described in a previous study(Bratulic et al. 2015). We used high-fidelity Phusion DNA polymerase to reduce the mutation rate during these PCR amplifications. Specifically, we performed a 14-cycle PCR using primers misFsmrt2/misRsmrt2 to amplify GFP variants from each replicate population (supplementary table S4, Supplementary Material online). A 30 µl PCR reaction consisted of 1 ng template plasmid, 1.5 U Phusion Hot Start II High-Fidelity DNA Polymerase (F-549L, Thermo Scientific), 400 µM dNTPs, 6 µl 5× Phusion HF Buffer, 0.9 µl 100% DMSO, and 400 nM primers misFsmrt2/misRsmrt2. We performed the PCR reaction with the following thermocycler program: 98 °C/30 s, 14 cycles of 98 °C/15 s, 70 °C/15 s and 72 °C/20 s, 72 °C/1 min. Subsequently, we added 5 U *Dpn*I and 5 U Exonuclease I (EN0581, Fermentas) to the PCR products, and incubated at 37 °C for 1 h to digest the template plasmid and the primers. After incubation at 80 °C for 20 min to inactivate these enzymes, we used the resulting PCR products as templates together with different barcode-tagged primers (supplementary table S4, Supplementary Material online) to perform barcoding PCRs. A unique 16-bp sequence is located at the 5-terminal of each barcode-tagged primer. We used a unique pair of one forward (F1-F18) and one reverse (R1-R18) barcode-tagged primers to barcode each replicate population from each generation of evolution. We performed the barcoding PCR amplification in a 50 µl PCR reaction by mixing 1 µl template, 2.5 U Phusion Hot Start II High-Fidelity DNA Polymerase, 400 µM dNTPs, 400 nM forward and reverse primers, 1.5 µl 100% DMSO, and 10 µl 5× Phusion HF Buffer. We performed the PCR reaction as follows: 98 °C/30 s, 29 cycles of 98 °C/15 s, 71 °C/15 s and 72 °C/20 s, 72 °C/5 min.

Subsequently, we used the QIAquick PCR purification kit to purify the barcode-tagged PCR products and checked the quality of amplicons by using a UV-Vis spectrophotometer (NanoDrop, Thermo Fisher Scientific) and through agarose gel electrophoresis. We measured the concentration of amplicons by using Qubit dsDNA BR Assay Kit (Q32853, Invitrogen). To detect potential errors that might occur during library preparation, we followed the same procedure to amplify the ancestral GFP gene. At the end, we pooled 20 ng DNA of each population from each generation of evolution into one tube and sent the resulting pool to the functional genomics center Zurich (FGCZ) for sequencing.

### Primary Data Analysis

We performed SMRT sequencing data analysis using the SMRT Link SMRT Link V9.0.0.92188 package. We used the protocol “Circular Consensus Sequences (CCS)” to assemble consensus reads from single-stranded subreads. To filter reads of GFP inserts, we set the full-pass subread number to ≥3, the predicted consensus accuracy to ≥0.99, and the insert length to 600–1,500 bp. We used the “Demultiplex Barcodes” application to demultiplex the resulting data by setting the “Minimum Barcode Score” to 80 and the “Filter Minimum Barcode Quality” to 26. We then mapped the demultiplexed reads to the ancestral GFP sequence using BLASR(Chaisson and Tesler 2012) by setting the mapped length to ≥700 bp and the mapping accuracy to ≥0.9. From the mapping data, we identified mapped reads that span the entire GFP coding region, and that had an average Phred quality above 20. This generated ∼500–2,000 reads for each replicate population during each generation (supplementary table S1, Supplementary Material online). We only used these reads for further analysis.

### Identification of Single Nucleotide Polymorphisms

We sequenced the ancestral GFP gene *gfp* and found that the major source of SMRT sequencing errors is single-nucleotide indels, which was also reported in a previous study (Laehnemann et al. 2016). In addition, because most indels result in nonfunctional variants, we ignored indels and only focused on analyzing point mutations. A mismatch of a GFP variant sequence to the ancestral GFP sequence was considered as a true single nucleotide polymorphism only if its Phred quality score was above 20. We identified single nucleotide polymorphisms and calculated their frequencies in each replicate population by using custom Python scripts (Python 2.7.12).

### Statistical Analysis

Unless specified otherwise, we used one-sided *t*-tests for statistical data analysis. We performed all statistical analysis using R version 4.0.3.

## Supplementary Material

Supplementary data are available at *Molecular Biology and Evolution* online.

## Supplementary Material

msab206_Supplementary_Data
