# Supplementary material for: Mistranslation Reduces Mutation Load in Evolving Proteins through Negative Epistasis with DNA Mutations
Source: Mol Biol Evol. 2021 Jul 13;38(11):4792–804. doi: 10.1093/molbev/msab206 (PMC8557407; doi:10.1093/molbev/msab206)
Supplement: msab206_Supplementary_Data [file msab206_supplementary_data.pdf]

Mistranslation reduces mutation load in evolving proteins through negative epistasis with DNA mutations

Jia Zheng, Ning Guo & Andreas Wagner*

Corresponding authors:
Andreas Wagner
Email: andreas.wagner@ieu.uzh.ch

This PDF file includes:

Figs. S1 to S8
Tables S1 to S4

Supplementary figures

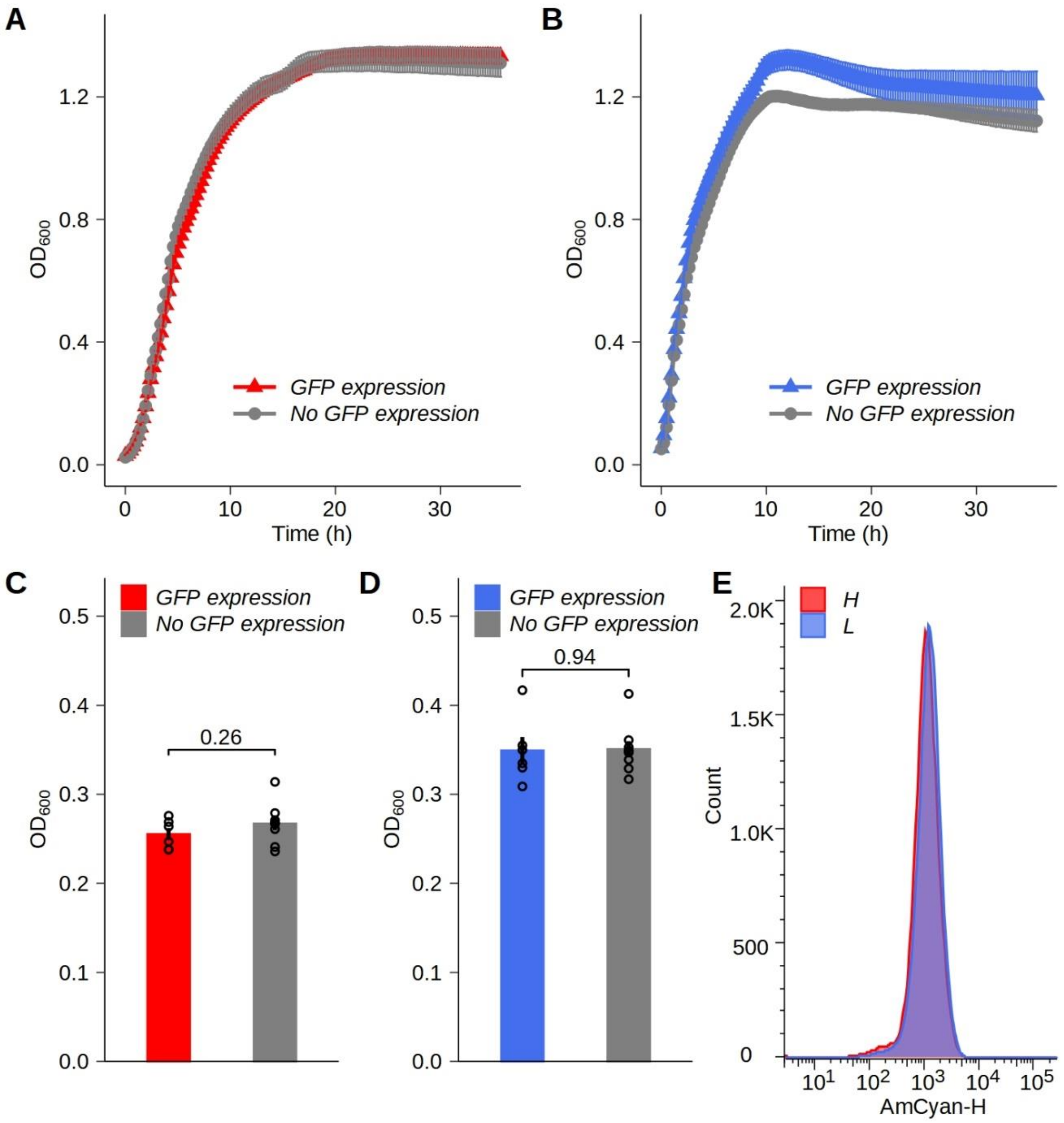

Fig. S1 Heterologous expression of GFP in high-mistranslation and low-mistranslation strains has little effect on strain growth.

(**A-B**) Growth curves of high-mistranslation **(A)** and low-mistranslation strains **(B)** with or without GFP expression**.** We grew both strains in 200 μL LB medium in a 96-well micro-plate for 36 hours in a TECAN

plate reader (37℃, 216 rpm), and recorded the OD600 values in ~15min intervals. Because some cells adhered to the bottom of their well in a 96-well micro-plate when OD600 values exceeded ~1.0, measurements after 10 hours of incubation may be inaccurate. **(C-D)** Cell densities of high-mistranslation (C) and low-mistranslation strains (D) with or without GFP expression after incubation for 36 hours. We grew both strains in 200 μL LB medium in a 96-well micro-plate in a TECAN Plate reader (37℃, 216 rpm), and measured cell densities of four-fold diluted cultures using a TECAN plate reader after 36 hours. **(E)** Distribution of fluorescence intensities of high-mistranslation (red) and low-mistranslation strains (blue) expressing GFP, after 24 h of incubation in 10-mL tubes (37℃, 220 rpm). Error bars represent one standard deviation based on at least six replicate populations in panels A-D. In panel E, the vertical axis indicates the number of cells at a given value of green fluorescence intensity (arbitrary units, horizontal axis). We performed two-sided t-tests to determine statistical significance in panels C-D. Corresponding *P* values are indicated above the bars. Our observations show that neither high-mistranslation nor low-mistranslation host strains experienced a substantial growth burden from expressing GFP. The strains also did not differ greatly in their GFP expression.

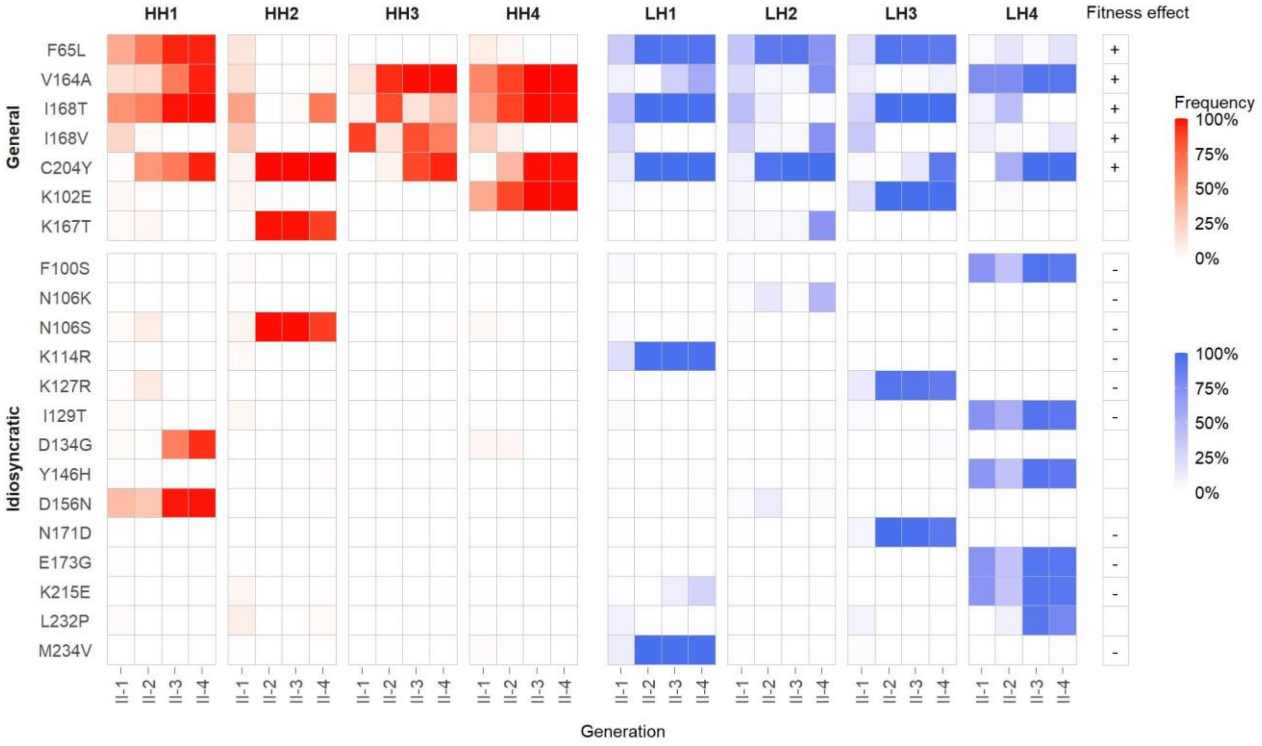

Fig. S2 Populations H→H accumulated fewer idiosyncratic mutations than populations L→H during phase II evolution.

The horizontal axes indicate time in generations of directed evolution, and the left vertical axis indicates the names of general and idiosyncratic mutations. See Figs. S3, S4 and S6 for how we determined the fitness effects of mutations on yellow fluorescence. Colored squares indicate variant frequencies (see color legend). The figure shows only those mutations that achieved a frequency exceeding 30% in at least one replicate population (*H→H* or *L→H*) at the end of Phase II. In the right-most column, beneficial and deleterious mutations are labeled with the symbols ‘+’ and ‘-’ (see details in Figs. S3, S4, and S5).

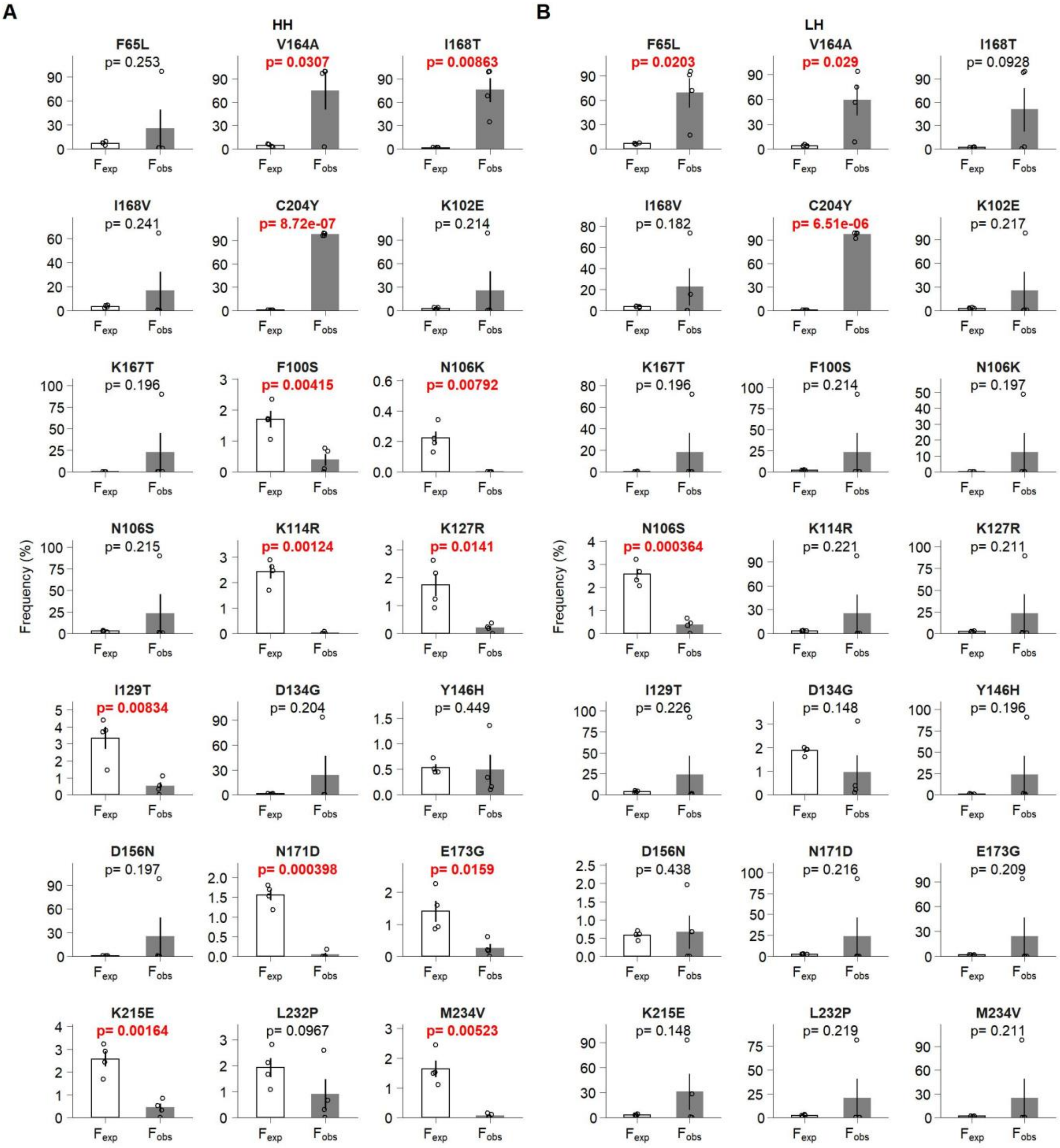
A
HH
F65L
p= 0.253
V164A
p= 0.0307
I168T
p= 0.00863
I168V
p= 0.241
C204Y
p= 8.72e-07
K102E
p= 0.214
K167T
p= 0.196
F100S
p= 0.00415
N106K
p= 0.00792
N106S
p= 0.215
K114R
p= 0.00124
K127R
p= 0.0141
I129T
p= 0.00834
D134G
p= 0.204
Y146H
p= 0.449
D156N
p= 0.197
N171D
p= 0.000398
E173G
p= 0.0159
K215E
p= 0.00164
L232P
p= 0.0967
M234V
p= 0.00523
Frequency (%)
F_{exp}
F_{obs}
B
LH
F65L
p= 0.0203
V164A
p= 0.029
I168T
p= 0.0928
I168V
p= 0.182
C204Y
p= 6.51e-06
K102E
p= 0.217
K167T
p= 0.196
F100S
p= 0.214
N106K
p= 0.197
N106S
p= 0.000364
K114R
p= 0.221
K127R
p= 0.211
I129T
p= 0.226
D134G
p= 0.148
Y146H
p= 0.196
D156N
p= 0.438
N171D
p= 0.216
E173G
p= 0.209
K215E
p= 0.148
L232P
p= 0.219
M234V
p= 0.211
Frequency (%)
F_{exp}
F_{obs}

Fig. S3 Estimating the fitness effects of twenty-one high-frequency mutations on yellow fluorescence by comparing the frequency of each of mutations after phase II evolution with its expected frequency under no selection.

Here we show the mutations that reached a frequency of >30% in at least one replicate population of *H*→*H* (A) and *L*→*H* (B) at the end of phase II evolution (see Fig. S2). We considered a mutation beneficial only if its frequency (F_{obs}, *observed frequency*) was significantly higher than the expected frequency (F_{exp}) in populations *H*→*H* or *L*→*H* at the end of phase II, whereas we consider a mutation deleterious when its frequency F_{obs} was significantly lower than the expected frequency F_{exp} (Please see details in the main text). Note that we considered the mutations F65L, V164A, I168T and C204Y as beneficial, because their frequencies (F_{obs}) were significantly or marginally higher than the expected frequencies (F_{exp}) after phase II evolution. In addition, although the frequency increase of the mutation I168V was not significantly higher than expected at the end of phase II, it was significantly higher than expected after the first generation of phase II (Fig. S4). The eventual decrease of its frequency in some replicate *H*→*H* and *L*→*H* populations at the end of phase II occurred because of clonal interference with the beneficial mutation I168T (Fig. S5). We thus considered the mutation I168V a beneficial mutation as well. We considered the mutations F100S, N106K, N106S, K114R, K127R, I129T, N171D, E173G, K215E and M234V as deleterious mutations, because their frequencies (F_{obs}) were significantly or marginally lower than the expected frequencies (F_{exp}) after phase II evolution. We performed one-sided t-tests to determine whether the frequency of each mutation (F_{obs}) was significantly lower or higher than its expected frequency (F_{exp}). Corresponding *P* values are indicated above the bars and those less than 0.05 are highlighted in red.

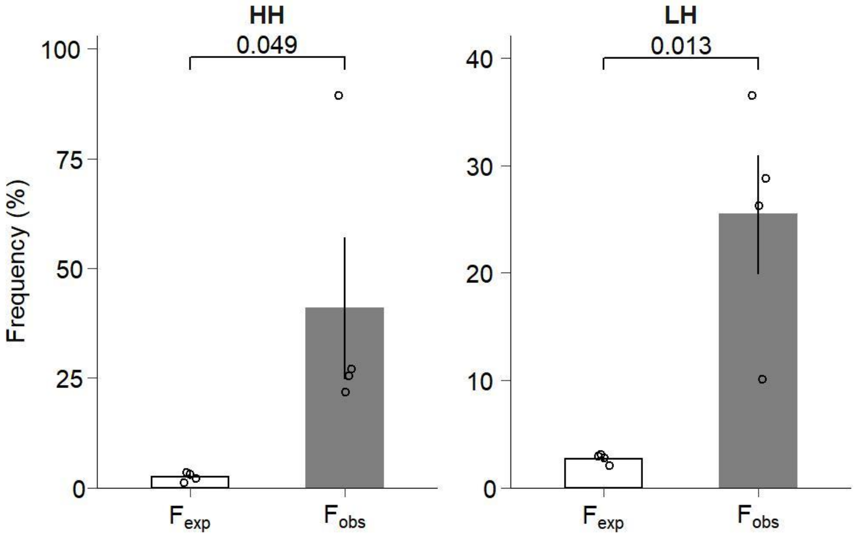

Fig. S4 The frequency of the mutation I168V was significantly higher than expected after the first generation of phase II in *H*→*H* populations and in *L*→*H* populations.

To generate the data for this figure, we first determined the frequency F_I of the variant I168V in populations *H* and *L* at the end of phase I. We then determined the expected frequency increase ΔF of this variant due to mutation pressure alone per generation of phase II from the sequence data of mutation libraries, i.e., libraries in which we had subjected ancestral GFP to mutation but no selection (see Methods). From these quantities, the expected frequency after one round of phase II evolution calculates as $F_{exp}= F_I+ 1\times\Delta F$. We calculated the observed frequency (F_{obs}) from the sequencing data of populations *H*→*H* and *L*→*H* after the first generation of phase II. Error bars indicate one standard deviation, based on four replicate populations (shown as small symbols). We performed one-sided t-tests to determine whether F_{obs} is significantly higher than F_{exp}. The resulting *P* values are indicated in each panel.

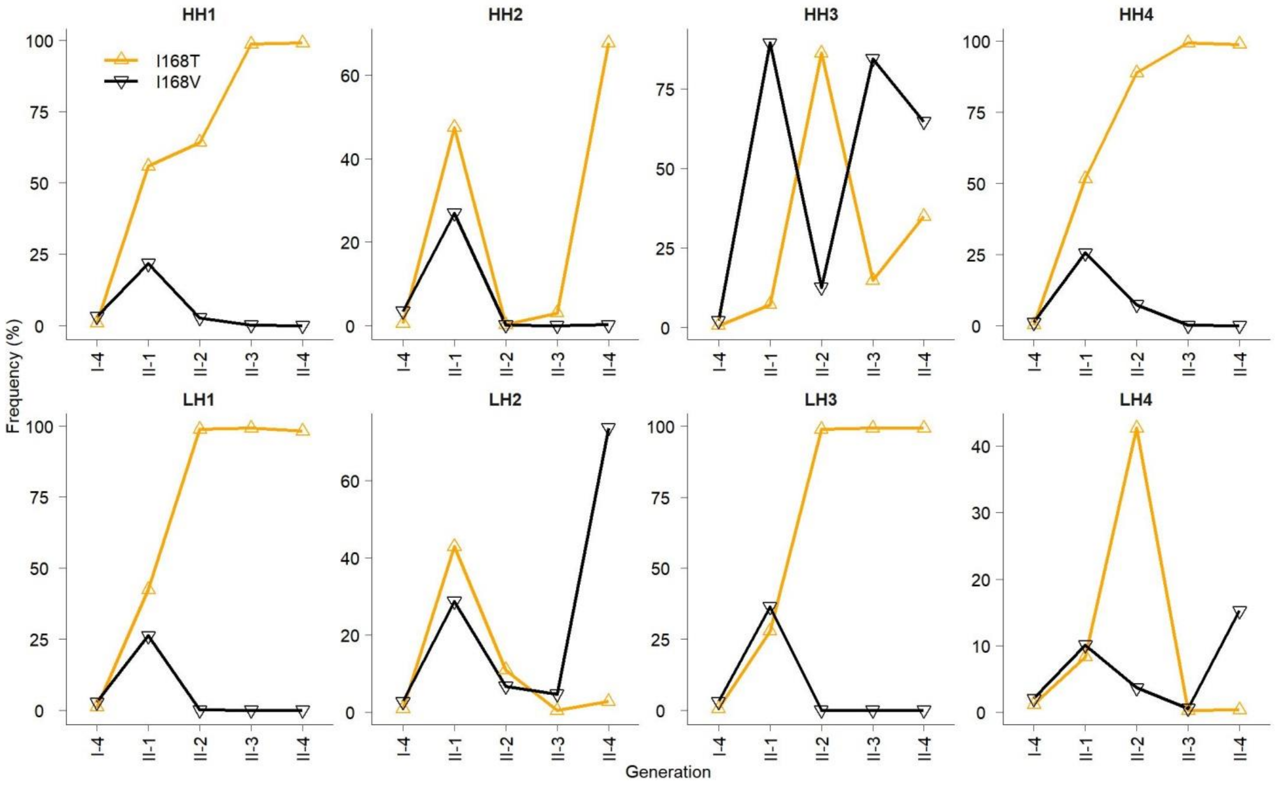

Fig. S5 Dynamics of the mutations I168T and I168V in each replicate *H*→*H* and *L*→*H* population during evolution.

The horizontal axes show time in generations of directed evolution. The vertical axes indicate the frequencies of the mutations I168T and I168V in the indicated *L*→*H* and *H*→*H* replicate populations. The frequencies of these mutations at the end of phase I (I-4) are also shown for *H* and *L* populations as the left-most data item in each panel. Although the frequency increase of the mutation I168V was not significantly higher than expected by mutation pressure alone at the end of phase II, it was significantly or marginally higher than expected in both populations *L*→*H* and *H*→*H* at the first generation of phase II (Fig. S4). The eventual decrease of its frequency in six out of eight replicate *H*→*H* and *L*→*H* populations at the end of phase II was likely caused by clonal interference with the beneficial mutation I168T. Based on the above observations, we considered the mutation I168V a beneficial mutation.

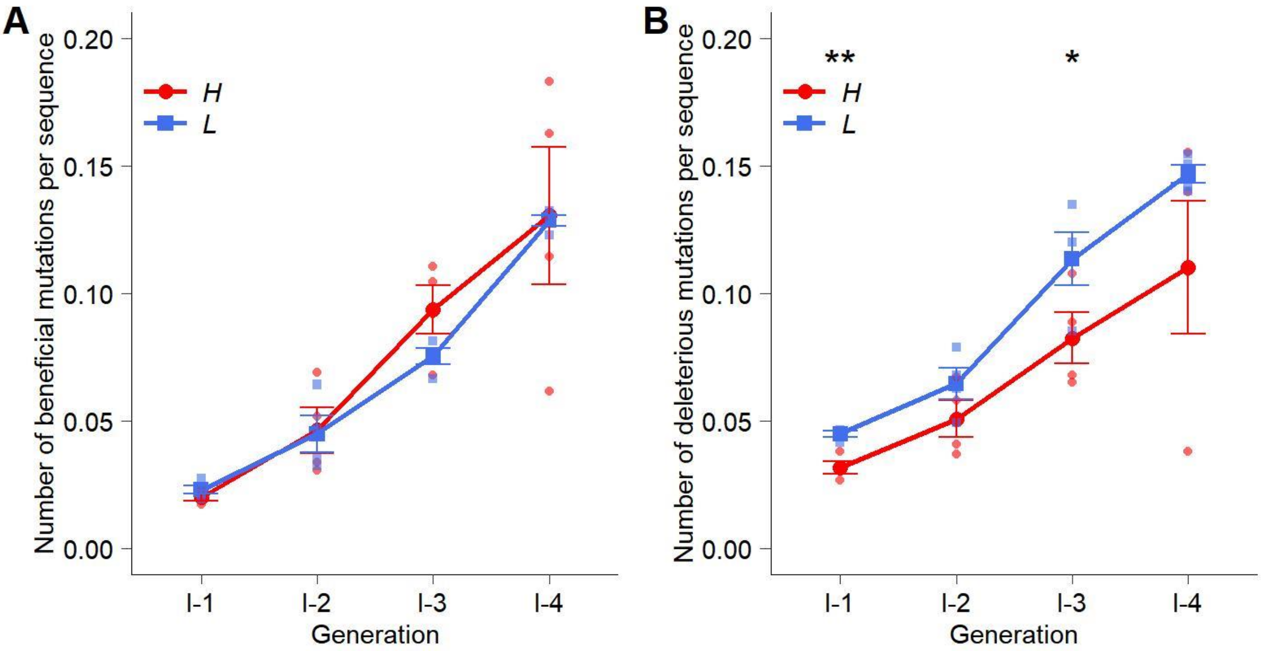

Fig. S6 Elevated mistranslation helps purge deleterious mutations.

(**A**) High-mistranslation populations *H* accumulated mutations beneficial for the new phenotype yellow fluorescence as rapidly as low-mistranslation populations *L* during phase I evolution. The horizontal axis shows time in generations of directed evolution. The vertical axis indicates the numbers of beneficial mutations per protein variant in *H* and *L* populations (see Figs. S3, S4, S5). The number of beneficial mutations in each generation was not significantly different between populations *H* and *L* during phase I evolution (P>0.1; One-sided T-tests). (**B**) High-mistranslation populations *H* accumulated fewer mutations with deleterious effects on the new phenotype yellow fluorescence than low-mistranslation populations *L* during phase I evolution. The horizontal axis shows time in generations of directed evolution. The vertical axis indicates the numbers of deleterious mutations per protein variant in *H* and *L* populations. Note that the figure is based on ten idiosyncratic mutations that are deleterious (see Fig. S2). These mutations are F100S, N106K, N106S, K114R, K127R, I129T, N171D, E173G, K215E, and M234V. We performed one-sided t-tests to determine whether the numbers of deleterious mutations per protein variant in each generation were

significantly smaller in populations *H* than in populations *L* during phase I evolution. * $P<0.05$, ** $P<0.01$.

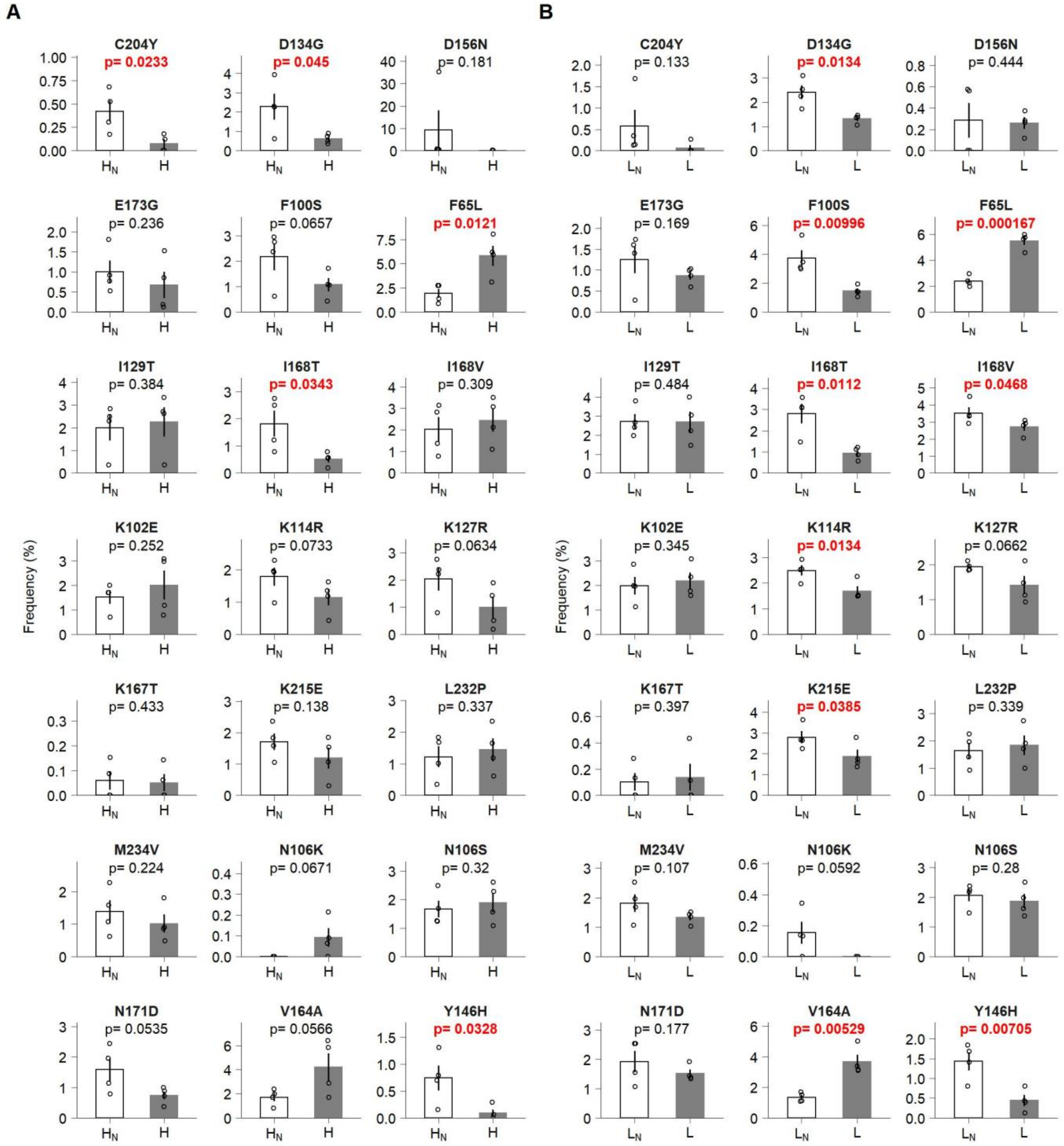
A
B
Frequency (%)
C204Y p= 0.0233
D134G p= 0.045
D156N p= 0.181
E173G p= 0.236
F100S p= 0.0657
F65L p= 0.0121
I129T p= 0.384
I168T p= 0.0343
I168V p= 0.309
K102E p= 0.252
K114R p= 0.0733
K127R p= 0.0634
K167T p= 0.433
K215E p= 0.138
L232P p= 0.337
M234V p= 0.224
N106K p= 0.0671
N106S p= 0.32
N171D p= 0.0535
V164A p= 0.0566
Y146H p= 0.0328
H_N
H
C204Y p= 0.133
D134G p= 0.0134
D156N p= 0.444
E173G p= 0.169
F100S p= 0.00996
F65L p= 0.000167
I129T p= 0.484
I168T p= 0.0112
I168V p= 0.0468
K102E p= 0.345
K114R p= 0.0134
K127R p= 0.0662
K167T p= 0.397
K215E p= 0.0385
L232P p= 0.339
M234V p= 0.107
N106K p= 0.0592
N106S p= 0.28
N171D p= 0.177
V164A p= 0.00529
Y146H p= 0.00705
L_N
L

Fig. S7 Estimating the fitness effects of twenty-one high-frequency mutations on green fluorescence by comparing the frequency of each of mutations between populations *H* and H_N, and between populations *L* and L_N at the end of phase I evolution.

The figure is based on mutations that reached a frequency exceeding 30% in at least one replicate *H*→*H* (A) and *L*→*H* (B) population at the end of phase II evolution (see Fig. S2). We performed one-sided t-tests to determine the significant difference in the frequency of each mutation between populations *H* and H_N and between populations *L* and L_N. The resulting *P* values are indicated in each panel above the bars. Error bars indicate one standard deviation, based on four replicate populations (shown as small symbols). The height of each bar shows the frequency of the mutation indicated at the top of the panel in the populations indicated on the horizontal axis (*H*, H_N, *L* and L_N) at the end of phase I evolution.

AAAGCGCTATTTCTTCCAGAATTGCC**CCCGTAGAAAAGATCAAAGGATCTTCTTGAGATCCTTTTTTTCT**
GCGCGTAATCTGCTGCTTGCAAACAAAAAAACCACCGCTACCAGCGGTGGTTTGTTTGCCGGATCAA
GAGCTACCAACTCTTTTTCCGAAGGTAACTGGCTTCAGCAGAGCGCAGATACCAAATACTGTCCTTC
TAGTGTAGCCGTAGTTAGGCCACCACTTCAAGAACTCTGTAGCACCGCCTACATACCTCGCTCTGCT
AATCCTGTTACCAGTGGCTGCTGCCAGTGGCGATAAGTCGTGTCTTACCGGGTTGGACTCAAGACGA
TAGTTACCGGATAAGGCGCAGCGGTCGGGCTGAACGGGGGGTTCGTGCACACAGCCCAGCTTGGA
GCGAACGACCTACACCGAACTGAGATACCTACAGCGTGAGCTATGAGAAAGCGCCACGCTTCCCGA
AGGGAGAAAGGCGGACAGGTATCCGGTAAGCGGCAGGGTCGGAACAGGAGAGCGCACGAGGGAGC
TTCCAGGGGGAAACGCCTGGTATCTTTATAGTCCTGTCGGGTTTCGCCACCTCTGACTTGAGCGTCG
ATTTTTGTGATGCTCGTCAGGGGGGCGGAGCCTATGGAAAAACGCCAGCAACGCGGCCTTTTTACG
GTTCCTGGCCTTTTGCTGGCCTTTTGCTCACATCCTAGTGCTTGGATTCTCACCAATAAAAAACGCCCG
GCGGCAACCGAGCGTTCTGAACAAATCCAGATGGAGTTCTGAGGTCATTACTGGATCTATCAACAGGAGTC
CAAGCGAGCTCTCGAACCCCAGAGTCCCGCTCAGAAGAACTCGTCAAGAAGGCGATAGAAGGCGATGCG
CTGCGAATCGGGAGCGGCGATACCGTAAAGCACGAGGAAGCGGTCAGCCCATTCGCCGCCAAGCTCTTCA
GCAATATCACGGGTAGCCAACGCTATGTCCTGATAGCGGTCCGCCACACCCAGCCGGCCACAGTCGATGAA
TCCAGAAAAGCGGCCATTTTCCACCATGATATTCGGCAAGCAGGCATCGCCATGGGTCACGACGAGATCCT
CGCCGTCGGGCATGCGCGCCTTGAGCCTGGCGAACAGTTCGGCTGGCGCGAGCCCCTGATGCTCTTCGTCC
AGATCATCCTGATCGACAAGACCGGCTTCCATCCGAGTACGTGCTCGCTCGATGCGATGTTTCGCTTGGTGG
TCGAATGGGCAGGTAGCCGGATCAAGCGTATGCAGCCGCCGCATTGCATCAGCCATGATGGATACTTTCTCG
GCAGGAGCAAGGTGAGATGACAGGAGATCCTGCCCCGGCACTTCGCCCAATAGCAGCCAGTCCCTTCCCG
CTTCAGTGACAACGTCGAGCACAGCTGCGCAAGGAACGCCCGTCGTGGCCAGCCACGATAGCCGCGCTGC
CTCGTCCTGCAGTTCATTCAGGGCACCGGACAGGTCGGTCTTGACAAAAAGAACCGGGCGCCCCTGCGCT
GACAGCCGGAACACGGCGGCATCAGAGCAGCCGATTGTCTGTTGTGCCCAGTCATAGCCGAATAGCCTCTC
CACCCAAGCGGCCGGAGAACCTGCGTGCAATCCATCTTGTTCAATCATGCGAAACGATCCTCATCCTGTCTC
TTGATCAGATCTTGATCCCCTGCGCCATCAGATCCTTGGCGGCAAGAAAGCCATCCAGTTTACTTTGCAGGG
CTTCCCAACCTTACCAGAGGGCGCCCCAGCTGGCAATTCCGACGTCTAAGAAACCATTATTATCATGACATT
AACCTATAAAAATAGGCGTATCACGAGGCCCTTTCGTCTTCAC*GGATCCCGGTAAGCGCATCCTCTCACGCCA*
CGAGACAACTTTCCGGGCTAAAAATTCACTCTAATTTGTATCATTAAGTAAATTTAGGATTAATCCTGGAACTTTTTTT
GTCGCCCAGCCAATGCTTTCAGTCGTGACTAATTTTCCTTGCGGAGGCTTGTCTGAAGCGGTTTCCGCGATTCTCT
TCTGTAAATTGTCGCTGACAAAAAAGATTAAACATACCTTATACAAGACTTTTTTTTCATATGCCTGACGGAGTTCAC
ACTTGTAAGTTTTCAACTACGTTGTAGACTTTACATCGCCAGGGGTGCTCGGCATAAGCCGAAGATATCGGTAGAG
TTAATATTGAGCAGATCCCCCGGTGAAGGATTTAACCGTGTTATCTCGTTGGAGATATTCATGGCGTATTTTGGATG
ATAACGAGGCGCAAAAAATGAAAAAGACAGCTATCGCGATTGCAGTGGCACTGGCTGGTCTCGCTACCGTAGCGC
*AGGCCGCTCCGAAAGATATCTCGATTTAAGAAGGAGATATACATCTCGAG*ATGATGAGCAAGGGCGAGGAGCTG
TTCACCGGGGTGGTGCCCATCCTGGTCGAGCTGGACGGCGACGTAAACGGCCACAAGTTCAGCGTGTCCG
GCGAGGGCGAGGGCGATGCCACCTACGGCAAGCTGACCCTGAAGTTCATCTGCACCACCGGCAAGCTGCC

CGTGCCCTGGCCCACCCTCGTGACCACCTTCAGCTACGGCCTGCAATGCTGCGCCCGCTACCCCGACCACA
TGAAGCTGCACGACTTCTTCAAGTCCGCCATGCCCGAAGGCTACGTCCAGGAGCGCACCATCTTCTTCAAG
GACGACGGCAACTACAAGACCCGCGCCGAGGTGAAGTTCGAGGGCGACACCCTGGTGAACCGCATCGAG
CTGAAGGGCATCGACTTCAAGGAGGACGGCAACATCCTGGGGCACAAGCTGGAGTACAACTACAACAGCC
ACAACGTCTATATCATGGCCGACAAGCAGAAGAACGGCATCAAGGTGAACTTCAAGATCCGCCACAACATC
GAGGACGGCAGCGTGCAGCTCGCCGACCACTACCAGCAGAACACCCCCATCGGCGACGGCCCCGTGCTGC
TGCCCGACAACCACTACCTGAGCTGCCAGTCCGCCCTGAGCAAAGACCCCAACGAGAAGCGCGATCACAT
GGTCCTGCTGGAGTTCGTGACCGCCGCCGGGATCACTCTCGGCATGGACGAGCTGTACAAGTGAAAGCTT
GGCATCAAATAAAACGAAAGGCTCAGTCGAAAGACTGGGCCTTTCGTTTTATCTGTTGTTTGTCGGTGAAC
GCTCTCCTGAGTAGGACAAATCCGCCGCCCTAGACCTAGGGTACGGGTTTTGCTGCCCGCAAACGGGCTGT
TCTGGTGTTGCTAGTTTGTTATCAGAATCGCAGATCCGGCTTCAGGTTTGCCGGCTG

Fig. S8 DNA sequence of the plasmid pXHO-*eGFP*. The pUC replication origin and the *pompA* promoter are highlighted in bold and italics, respectively. The *gfp* gene is shown in green.

Supplementary tables

Table S1 Number of reads sequenced by SMRT sequencing for evolving populations during evolution

Populations	Reads in each generation							
	I1	I2	I3	I4	II1	II2	II3	II4
H_N1	1092	935	412	1141				
H_N2	252	809	714	655				
H_N3	1237	874	321	765				
H_N4	1116	1193	367	884				
H1	1559	2002	1404	1210				
H2	1160	1186	1567	1114				
H3	1368	1860	2180	1606				
H4	1808	1657	1313	1147				
H→H1					1308	1460	1739	1304
H→H2					809	1436	1650	815
H→H3					1879	1520	1950	593
H→H4					1561	1397	2224	979
L_N1	1399	775	397	712				
L_N2	508	616	161	357				
L_N3	1134	932	365	752				
L_N4	1296	1114	382	869				
L1	1464	1765	2356	1515				
L2	1483	1368	2212	1614				
L3	1018	1174	1393	876				
L4	1637	1438	2349	1418				
L→H1					1511	2144	900	1871
L→H2					1646	1903	2236	1068
L→H3					1020	1243	875	767
L→H4					1742	1447	2175	1100
Mut_lib [a]	1227	687	1087	939	1349	1009	1498	1770
GFP (Ancestor) [b]	1163							
GFP (Ancestor) [b]	2285							

[a] To estimate the mutation rate, we used our mutagenesis protocol in each generation to create a library of mutants of

ancestral GFP, and sequenced this library before selection (see Methods).

[b] We used unmutated GFP to estimate the sequencing error during SMRT sequencing (see Methods).

Table S2 Mean number of SNPs per GFP molecule for evolving populations during each generation determined by SMRT sequencing.

Populations	Mean number of SNPs per GFP molecule							
	I1	I2	I3	I4	II1	II2	II3	II4
H_N1	1.641	3.659	5.316	7.886				
H_N2	1.685	3.549	5.333	7.702				
H_N3	1.669	3.641	5.206	7.787				
H_N4	1.640	3.358	5.038	7.583				
H1	0.638	1.351	2.141	2.894				
H2	0.672	1.417	2.211	3.073				
H3	0.723	0.890	1.569	2.296				
H4	0.679	0.936	1.648	1.517				
H→H1					4.687	5.354	7.604	8.312
H→H2					5.692	4.030	4.086	5.571
H→H3					6.436	4.711	4.352	5.170
H→H4					4.850	6.457	6.023	6.335
L_N1	1.649	3.499	5.229	7.552				
L_N2	1.565	3.589	5.199	7.768				
L_N3	1.671	3.733	4.932	7.362				
L_N4	1.629	3.491	5.246	7.436				
L1	0.781	0.999	1.846	2.789				
L2	0.786	1.567	2.324	3.212				
L3	0.699	1.561	2.456	3.382				
L4	0.779	1.386	2.293	3.025				
L→H1					4.897	9.840	11.333	10.553
L→H2					4.325	4.602	4.160	7.583
L→H3					4.482	7.909	8.305	9.072
L→H4					5.535	6.863	8.010	8.677
Mut_lib [a]	1.7	2.006	2.037	1.993	0.671	0.705	0.612	0.784
GFP (Ancestor) [b]	0.025							
GFP (Ancestor) [b]	0.035							

[a] To estimate the mutation rate, we used our mutagenesis protocol in each generation to create a library of mutants of

ancestral GFP, and sequenced this library before selection (see Methods).

[b] We used GFP as a control to estimate the sequencing error during SMRT sequencing (see Methods).

Table S3 Mean number of amino-acid changes per GFP molecule for evolving YFP populations during each generation.

Populations	Mean number of amino-acid changes per GFP molecule							
	I1	I2	I3	I4	II1	II2	II3	II4
H_N1	1.395	3.027	4.439	6.158				
H_N2	1.440	2.937	4.520	6.266				
H_N3	1.370	3.018	4.268	6.396				
H_N4	1.372	2.599	3.967	6.129				
H1	0.434	0.887	1.377	1.898				
H2	0.416	0.935	1.444	1.978				
H3	0.497	0.573	0.983	1.481				
H4	0.427	0.626	1.143	1.142				
H→H1					3.317	3.752	5.625	6.182
H→H2					4.354	3.025	3.078	4.384
H→H3					4.611	3.178	3.251	3.966
H→H4					3.439	4.395	4.023	4.203
L_N1	1.370	2.903	4.476	6.319				
L_N2	1.301	3.013	4.323	6.532				
L_N3	1.446	3.141	4.055	6.157				
L_N4	1.386	2.933	4.351	6.223				
L1	0.551	0.686	1.256	1.900				
L2	0.562	1.060	1.576	2.190				
L3	0.484	1.075	1.640	2.243				
L4	0.555	0.937	1.577	2.027				
L→H1					3.385	4.971	5.772	6.503
L→H2					3.211	3.584	3.161	5.966
L→H3					3.429	4.924	5.266	6.020
L→H4					5.116	5.836	7.853	8.302
Mut_lib [a]	1.687	1.422	1.691	1.646	0.567	0.606	0.520	0.650
GFP (Ancestor) [b]	0.016							
GFP (Ancestor) [b]	0.021							

[a] To estimate the mutation rate, we used our mutagenesis protocol in each generation to create a library of mutants of

ancestral GFP, and sequenced this library before selection (see Methods). [b] We used GFP as a control to estimate the sequencing error during SMRT sequencing (see Methods).

Table S4 Primers for barcoding PCR

Primers		Sequence
Forward primers	1F	CAGCAGATCATGTCGAgcagtcgaacatgtagctgactcaggtcac
	2F	TACGATCGTAGCTGCTgcagtcgaacatgtagctgactcaggtcac
	3F	GTACACGCTGTGACTAgcagtcgaacatgtagctgactcaggtcac
	4F	ACAGTGCGCTGTCTATgcagtcgaacatgtagctgactcaggtcac
	5F	TATCAGCACGACATGCgcagtcgaacatgtagctgactcaggtcac
	6F	ACTGCGAGATACACACgcagtcgaacatgtagctgactcaggtcac
	7F	CATACATCGCGCAGTAgcagtcgaacatgtagctgactcaggtcac
	8F	CACATATCAGAGTGCGgcagtcgaacatgtagctgactcaggtcac
	9F	GATAGCTGCTAGCTGAgcagtcgaacatgtagctgactcaggtcac
	10F	TCACTGTGTGTGTCTGgcagtcgaacatgtagctgactcaggtcac
	11F	GATCTGTCGTGAGCGTgcagtcgaacatgtagctgactcaggtcac
	12F	TATCTGAGCGCGAGCAgcagtcgaacatgtagctgactcaggtcac
	13F	CTCACGTACGTCACACgcagtcgaacatgtagctgactcaggtcac
	14F	CGTGTCGCGCATATCTgcagtcgaacatgtagctgactcaggtcac
	15F	TGTGCACGACAGCAGTgcagtcgaacatgtagctgactcaggtcac
	16F	ACGTCAGCACTGCTCTgcagtcgaacatgtagctgactcaggtcac
	17F	GACTGCACATGCACGAgcagtcgaacatgtagctgactcaggtcac
	18F	CTACGAGACAGATCGCgcagtcgaacatgtagctgactcaggtcac
Reverse primers	1R	CGAGTAGACAGTGACTtggatcacttgtgcaagcatcacatcgtag
	2R	AGTGCACGAGCATATGtggatcacttgtgcaagcatcacatcgtag
	3R	CGTGTGATGTCTACAGtggatcacttgtgcaagcatcacatcgtag
	4R	ACACGCGATCTAGTGTtggatcacttgtgcaagcatcacatcgtag
	5R	AGCGCAGTGTATAGTGtggatcacttgtgcaagcatcacatcgtag
	6R	GATGAGAGAGCTCTCTtggatcacttgtgcaagcatcacatcgtag
	7R	TATGCAGTCTGTCGTCtggatcacttgtgcaagcatcacatcgtag
	8R	TCACGCTCTGTCTACTtggatcacttgtgcaagcatcacatcgtag
	9R	GCGATCTAGCTATGTCtggatcacttgtgcaagcatcacatcgtag
	10R	GCTATACGCTCGATACtggatcacttgtgcaagcatcacatcgtag
	11R	GTGACTGCGTGTCTAGtggatcacttgtgcaagcatcacatcgtag
	12R	CGAGTGCTAGACGATGtggatcacttgtgcaagcatcacatcgtag
	13R	ACGCACTATGACGTCGtggatcacttgtgcaagcatcacatcgtag
	14R	CTGTCAGAGTAGCTCGtggatcacttgtgcaagcatcacatcgtag
	15R	TCGCGATAGTCTCGCAtggatcacttgtgcaagcatcacatcgtag

16R	GATGCTCGAGTCGATCtggatcacttgtgcaagcatcacatcgtag
17R	GTGAGCGCAGTGAGTAtggatcacttgtgcaagcatcacatcgtag
18R	ACAGATGTCTGTGCGCtggatcacttgtgcaagcatcacatcgtag
misFsmrt2	gcagtcgaacatgtagctgactcaggtcacTAGCGCAGGCCGCTCCGA
misRsmrt2	tggatcacttgtgcaagcatcacatcgtagCGACTGAGCCTTTCGTTTTATTTGATGC

Sequences shown in upper case refer to barcoding regions, except for the primers misFsmrt2/misRsmrt2, where they refer to two DNA sequences that are located in flanking regions of *gfp* gene.
